# Bacterial community assembly driven by temporal succession rather than spatial heterogeneity in Lake Bosten: a large lake suffering from eutrophication and salinization

**DOI:** 10.3389/fmicb.2023.1261079

**Published:** 2023-09-20

**Authors:** Hao Liu, Jiangyu Dai, Ziwu Fan, Bei Yang, Hang Wang, Yang Hu, Keqiang Shao, Guang Gao, Xiangming Tang

**Affiliations:** ^1^State Key Laboratory of Hydraulics and Mountain River Engineering, Sichuan University, Chengdu, China; ^2^Key Laboratory of Taihu Basin Water Resources Management, Ministry of Water Resources, Nanjing Hydraulic Research Institute, Nanjing, China; ^3^State Key Laboratory of Lake Science and Environment, Nanjing Institute of Geography and Limnology, Chinese Academy of Sciences, Nanjing, China; ^4^Key Laboratory of Agricultural Environment of the Lower Reaches of the Yangtze River, Institute of Agricultural Resources and Environment, Jiangsu Academy of Agricultural Sciences, Nanjing, China

**Keywords:** Lake Bosten, oligosaline, bacterial community structure, cooccurrence network, assembly mechanism

## Abstract

Oligosaline lakes in arid and semi-arid regions play a crucial role in providing essential water resources for local populations. However, limited research exists on the impact of the environment on bacterial community structure in these lakes, co-occurrence patterns and the mechanisms governing bacterial community assembly. This study aims to address this knowledge gap by examining samples collected from five areas of Lake Bosten over four seasons. Using the 16S rRNA gene sequencing method, we identified a total of 510 to 1,005 operational taxonomic units (OTUs) belonging to 37 phyla and 359 genera in Lake Bosten. The major bacterial phyla were Proteobacteria (46.5%), Actinobacteria (25.9%), Bacteroidetes (13.2%), and Cyanobacteria (5.7%), while the major genera were *hgcI_clade* (12.9%), *Limnohabitans* (6.2%), and *Polynucleobacter* (4.7%). Water temperature emerged as the primary driver of these community structure variations on global level. However, when considering only seasonal variations, pH and nitrate were identified as key factors influencing bacterial community structures. Summer differed from other seasons in aspects of seasonal symbiotic patterns of bacterial communities, community assembly and function are different from other seasons. There were notable variations in bacterial community structures between winter and summer. Deterministic processes dominated community assembly, but there was an increase in the proportion of stochastic processes during summer. In summer, the functions related to photosynthesis, nitrogen fixation, and decomposition of organic matter showed higher abundance. Our findings shed light on the response of bacterial communities to environmental changes and the underlying mechanisms of community assembly in oligosaline lakes in arid regions.

## 1. Introduction

Arid and semi-arid regions, characterized by average annual precipitation between 25 and 500 mm, encompass approximately one-third of the world’s land area and are inhabited by nearly 400 million people. Freshwater lakes in these regions serve as vital water resources on which local populations depend for their survival (Williams, 1999). However, the distribution of rainfall and temperature has been altered due to the effects of global climate change, resulting in a significant reduction in the water surface area of inland northwest China (Li et al., 2007). Moreover, the discharge of pollutants into these water bodies has led to increased salinity and eutrophication, posing threats not only to the livelihoods of local residents but also to the ecological environment within the lake basin (Wang and Qin, 2017; Fang et al., 2018). Consequently, lakes situated in arid and semi-arid regions are considered early indicators of regional and global environmental changes, with their resident bacteria recognized as highly responsive to these alterations (Adrian et al., 2009; Williamson et al., 2009; Tang et al., 2015).

Heterotrophic bacteria play a crucial role in biogeochemical processes within aquatic ecosystems (Cotner and Biddanda, 2002). As decomposers, they are involved in micro-pollutant degradation (Johnson et al., 2015) and contribute to the maintenance of ecosystem stability (Awasthi et al., 2014). Therefore, understanding the composition, structure, and response of bacterial communities in water bodies to environmental changes becomes imperative. Numerous studies have focused on the composition, structure, and response of bacterial communities in lakes, particularly in shallow eutrophic lakes such as Lake Taihu (Niu et al., 2015; Kong et al., 2018; Xie et al., 2020). Additionally, research has been conducted on lakes in the Qinghai-Tibet Plateau (Liu et al., 2014, 2017; Ren et al., 2022), dammed or stepped reservoirs (Chen et al., 2020; Xie et al., 2021), and temperate or cold lakes (Yannarell et al., 2003; Daniel et al., 2016).

In recent years, there has been a growing interest in studying the mechanisms of community assembly, driven by the recognition that stochastic processes may significantly influence community dynamics. The balance between community succession based on niche theory and neutral theory is currently a central research focus in microbial ecology (Dini-Andreote et al., 2015). While studies have explored community assembly processes in eutrophic lakes on different scales of time and space (Zhao et al., 2017; Liu et al., 2020), and under different environmental gradients (Aguilar and Sommaruga, 2020), fewer investigations have been conducted in oligosaline lakes with less trophic levels.

Lake Bosten, situated in the central Tianshan Mountains, is the largest inland freshwater lake in China, covering an area of 1,064 km^2^ (1,047 m a.s.l.) with an average water depth of 8 m. The annual evaporation in the Lake Bosten area is 1881 mm (Bai et al., 2011). Over the past 50 years, Lake Bosten has undergone significant changes due to climate shifts and human activities (Yao et al., 2018). Salinity has increased from 0.38 g/L to 1.46 g/L, and nutrient levels have transitioned from oligotrophic to mesotrophic (Tang et al., 2015). These ongoing transformations provide a unique opportunity to study the response of microbial communities to salinization and eutrophication.

While progress has been made in understanding the seasonal changes in bacterial community structure in the sediment of Lake Bosten (Zhang et al., 2020), surface water studies have been limited to single-season investigations (Tang et al., 2012, 2015). Furthermore, there is a scarcity of research on the temporal and spatial variations in bacterial communities in the surface water column, which represents the interface layer between the water body and the external environment (Song et al., 2018). Additionally, little is known about the mechanisms of bacterial community assembly in oligosaline water habitats in arid regions. In this study, we employed high-throughput sequencing technology, redundancy analysis, co-occurrence network analysis, and neutral community models to explore the spatiotemporal changes in bacterial communities in Lake Bosten, their relationship with environmental factors, and the mechanisms governing bacterial assembly. Our specific objectives are as follows: (1) to identify the key environmental factors driving the spatiotemporal heterogeneity of bacterial communities in the surface water of Lake Bosten under the pressures of eutrophication and salinization, and (2) to investigate the potential bacterial community assembly mechanisms, community stability, and ecological function.

## 2. Materials and methods

### 2.1. Study area and sampling procedures

**Figure 1:**
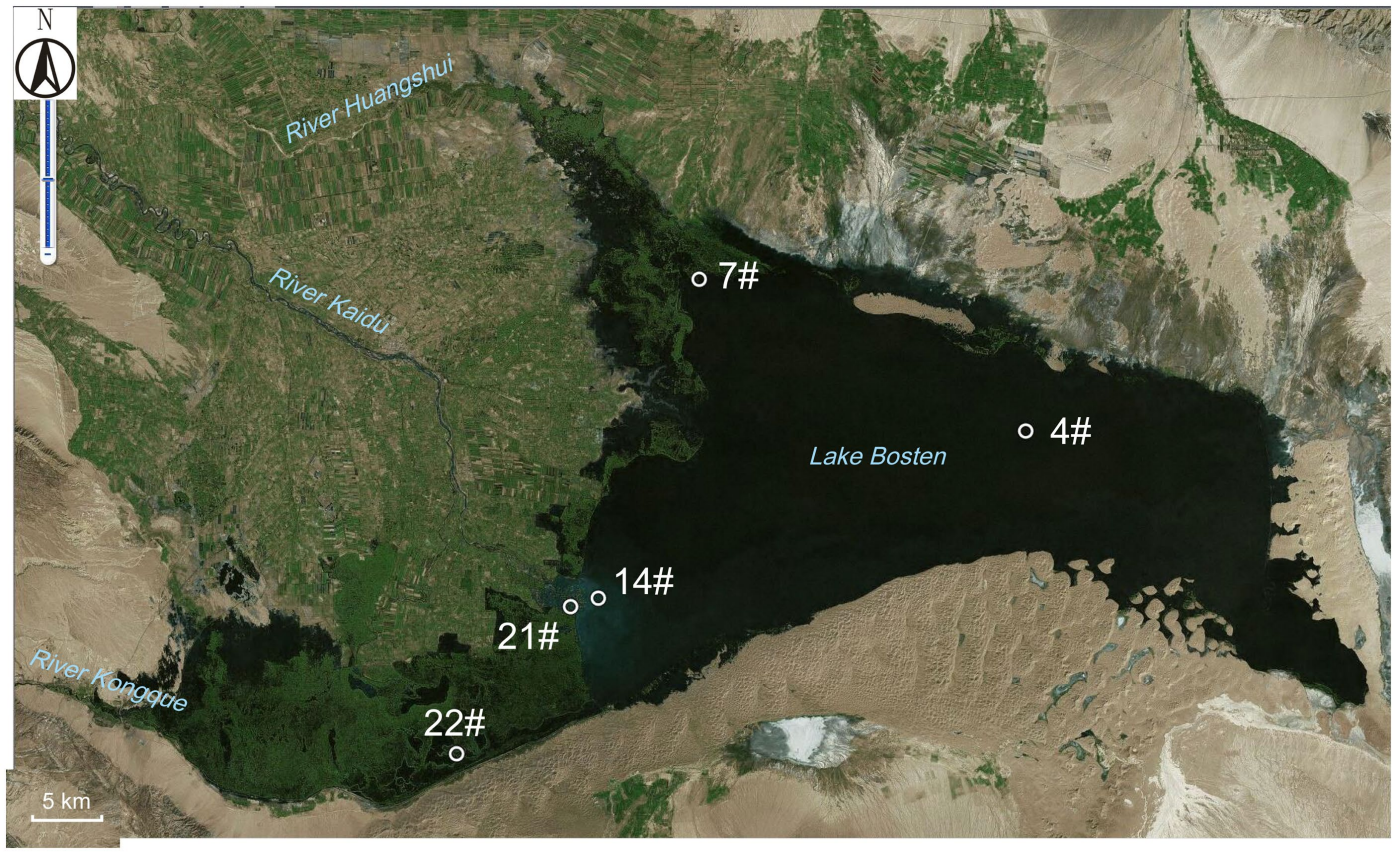
The sampling sites within Lake Bosten. The specific coordinates and descriptions of each site are as follows: Site 4# (87.1333° E, 42.0000° N): Located in the middle of Lake Bosten. Site 7# (86.8417° E, 42.1000° N): This site is situated in the estuary of River Huangshui, which is a seasonal river affected by agricultural pollution and other human activities. Site 14# (86.7417° E, 41.8889° N): Found at the estuary of River Kaidu, the largest perennial river with the highest annual volume of water. Site 21# (86.7269° E, 41.8836° N): Positioned near the boundary between the lake and the swamp area. Site 22# (86.6247° E, 41.7858° N): Located within the swamp area of Lake Bosten. These sampling sites were chosen to capture different environmental conditions and potential sources of influence within the lake.

A total of 20 surface water samples were collected from Lake Bosten between 2010 and 2011. These samples were obtained from five different sampling sites, as illustrated in Figure 1. The collection of water samples followed a seasonal schedule, with undisturbed duplicate water columns being collected in August 2010 (summer), October 2010 (fall), January 2011 (winter), and May 2011 (spring). To preserve the integrity of the samples, they were transferred to sterile containers immediately after collection. Subsequently, subsamples of 500–1,000 mL from each water column were filtered through 0.2 μm polycarbonate filters (Millipore) in the laboratory, using a vacuum pump. The filtered samples were carefully stored at −80°C until DNA extraction for 16S rRNA gene analysis. The remaining water samples were transported to the laboratory for chemical analysis.

### 2.2. Physicochemical analysis

Water depth (WD) and transparency (SD) were measured *in situ* using a water depth meter (Uwitec, Austria) and a Secchi disk (BP21-SD-20), respectively. Water temperature (WT), pH, turbidity (TUB), total dissolved solids (TDS), salinity (Sal) and dissolved oxygen (DO) were measured *in situ* with a multiparameter water quality probe (YSI 6600 v2, Yellow Springs Instruments Inc., United States). Concentrations of total nitrogen (TN), total nitrate-nitrogen (NO_3_^−^), ammonia-nitrogen (NH_4_^+^), total phosphorus (TP), chlorophyll a (Chl-*a*), chloride (Cl^−^), sulfate (SO_4_^2−^), and total organic carbon (TOC) were determined in the laboratory using standard methods (Jin and Tu, 1990). Bacterial abundance (BA) was enumerated using epifluorescence microscopy, following the method outlined by Tang et al. (2015).

### 2.3. DNA extraction purification and PCR amplification

DNA extraction from the collected water samples was performed using the FastDNA^®^ Spin Kit for Soil (MP Biomedicals). The integrity of the extracted DNA was assessed by subjecting it to 0.8% agarose gel electrophoresis. Gel electrophoresis was conducted at a voltage of 120 V for approximately 30 min.

For PCR amplification, the V1-V3 region of the bacterial 16S rRNA gene was targeted using universal primers 8 F (5’-AGAGTT TGATCCTGGCTCAG-3′) and 533 R (5’-TTACCGCGGCTG CTGGCAC-3′) (Bai et al., 2012). Duplicate PCR products were purified and concentrated using the E.Z.N.A.^®^ Cycle-Pure Kit. The purified products from each sample were then combined in equimolar ratios to create a mixed sample for 454 pyrosequencing.

### 2.4. 454 pyrosequencing and data analysis

Amplicon pyrosequencing was carried out on a Roche Genome Sequencer GS FLX Titanium platform using a 454/Roche A sequencing primer kit. High-quality sequences were obtained by removing the primers, and labels, and performing quality control procedures. The resulting sequences were then subjected to clustering using UCLUST, implemented in QIIME2 2018.11 software, with a minimum identity threshold of 97% to generate operational taxonomic units (OTUs) (Caporaso et al., 2010). Representative sequences for each OTU were trimmed and compared against the Bacterial Silva database (version 138) using QIIME.

Statistical analyses and data visualization were performed using *ggplot2* package in R 4.1.3.^1^ Non-metric multidimensional scaling (nMDS) was performed using the metaMDS function based on the Bray-Curtis distance calculated by the vegdist function after Hellinger transformation. Analysis of similarity (ANOSIM) was used to assess the significance of temporal and spatial differences in bacterial communities (Clarke, 1993). Redundancy analysis (RDA) was carried out by the *vegan* package (Borcard et al., 2011). The relationship between OTUs, functional community compositions, and each environmental factor was examined using partial (geographic distance corrected) mantel tests, performed with the *ggcor* package (Sunagawa et al., 2015). Kruskal-Wallis tests was used to calculate the significant differences between groups.

The functional prediction analysis was performed on the Tutools platform^2^ based on the FAPROTAX database, an online data analysis website that provides free access to its services. The Tutools platform was also used to generate a bubble chart visualizing the functions.

### 2.5. Co-occurrence network analysis of bacterial communities

To analyze the co-occurrence patterns of bacterial communities in Lake Bosten, Spearman’s correlation coefficients (*r*) were calculated using the *psych* package in R 4.1.3 (Barberan et al., 2014; Codello et al., 2023). The resulting correlation coefficients were used to construct co-occurrence networks. The topological properties of the networks, such as robustness and vulnerability, were evaluated using the *igraph* package in R 4.1.3 (Yuan et al., 2021; Shen et al., 2022). To identify key species in the co-occurrence network, thresholds based on degree, closeness centrality, and betweenness centrality were used. Species with a degree greater than 40, closeness centrality greater than 0.44, and betweenness centrality less than 0.12 are considered key species in the network (Banerjee et al., 2019). The resulting networks were visualized using Gephi v0.9, an interactive platform for network visualization.

### 2.6. Neutral community model

In the study, Sloan’s community model was utilized to examine the potential influence of stochastic processes on the phytoplankton community (Sloan et al., 2006). This model provides insights into the relationship between observed species abundance in local communities and their relative abundance in the larger metacommunity. It is based on neutral theory and can be applied to large microbial populations. The neutral model was implemented using the *MicEco* package, which provides tools for ecological analysis in R 4.1.3.

### 2.7. Deposition of nucleotide sequence accession numbers

The raw bacterial 16S rRNA gene sequences determined in the present study have been deposited in the Genome Sequence Archive of the BIG Data Center^3^ under the accession number CRA011126.

## 3. Results

### 3.1. Spatial and temporal variation in physicochemical parameters

WT ranged widely across the four seasons, from 1.23°C to 23.96°C, indicating significant seasonal fluctuations. The average DO concentration in winter (12.1 ± 4.7 mg/L) was significantly higher than that in summer, suggesting a seasonal difference in DO levels. The concentration of TOC in spring (17.8 ± 4.2 mg/L) was significantly higher than that in fall and winter, where it remained below 10 mg/L. Chl-*a* concentration was higher in fall (4.0 ± 2.3 μg/L) compared to winter (1.7 ± 0.7 μg/L), indicating a significant seasonal variation. The mean TP concentration in spring (0.06 mg/L) was higher compared to the other three seasons.

The highest SD was observed in the center of Lake Bosten (4#), reaching 3.65 ± 0.68 m, which was significantly higher than other sampling sites. SD in the estuary of River Huangshui (7#) and the swamp area of Lake Bosten (22#) were also significantly higher compared to the estuary of River Kaidu (14#) and the boundary between lake and swamp area (21#). The variation pattern of turbidity was similar to SD, with higher levels observed in the center of the lake (4#) and in the estuary of River Huangshui (7#) and swamp area (22#). pH at sites 21# and 22# was lower compared to other sites. DO at site 21# was extremely low (1.5 ± 1.7 mg/L), while the concentration of NH_4_^+^ at this site was higher than at other sites (0.59 ± 0.31 mg/L), especially compared to site 4#. TN concentration at site 22# was notably lower than at site 21# (Supplementary Table S1, Supplementary Figure S1).

### 3.2. Profiles of bacterial communities in Lake Bosten

The number of high-quality sequences obtained varied from 14,323 to 23,593 across different samples, with an average of 18,051. The number of OTUs varied from 510 to 1,005, with an average of 742. The rarefaction curves reached a plateau after the sequencing depth of 15,000, indicating that the sequencing depth was sufficient to capture a robust diversity of bacterial communities (Supplementary Figure S2).

Bacterial community in Lake Bosten contained a total of 37 phyla. The major phyla in Lake Bosten were Proteobacteria (46.5%), Actinobacteria (25.9%), Bacteroidetes (13.2%), and Cyanobacteria (5.7%). The relative proportions of these dominant phyla varied across seasons and sampling sites. Proteobacteria accounted for a higher proportion (more than 60%) of the bacteria at sites 22# and 14# in spring but was lower (approximately 40%) at sites 4# and 7#. The relative proportion of cyanobacteria increased at site 7# compared to other sites. In summer, there was an increase in the proportion of Firmicutes at sites 7# and 14#, potentially due to their locations at the estuary of River Huangshui and River Kaidu, respectively. The proportion of Bacteroidetes increased in winter.

**Figure 2:**
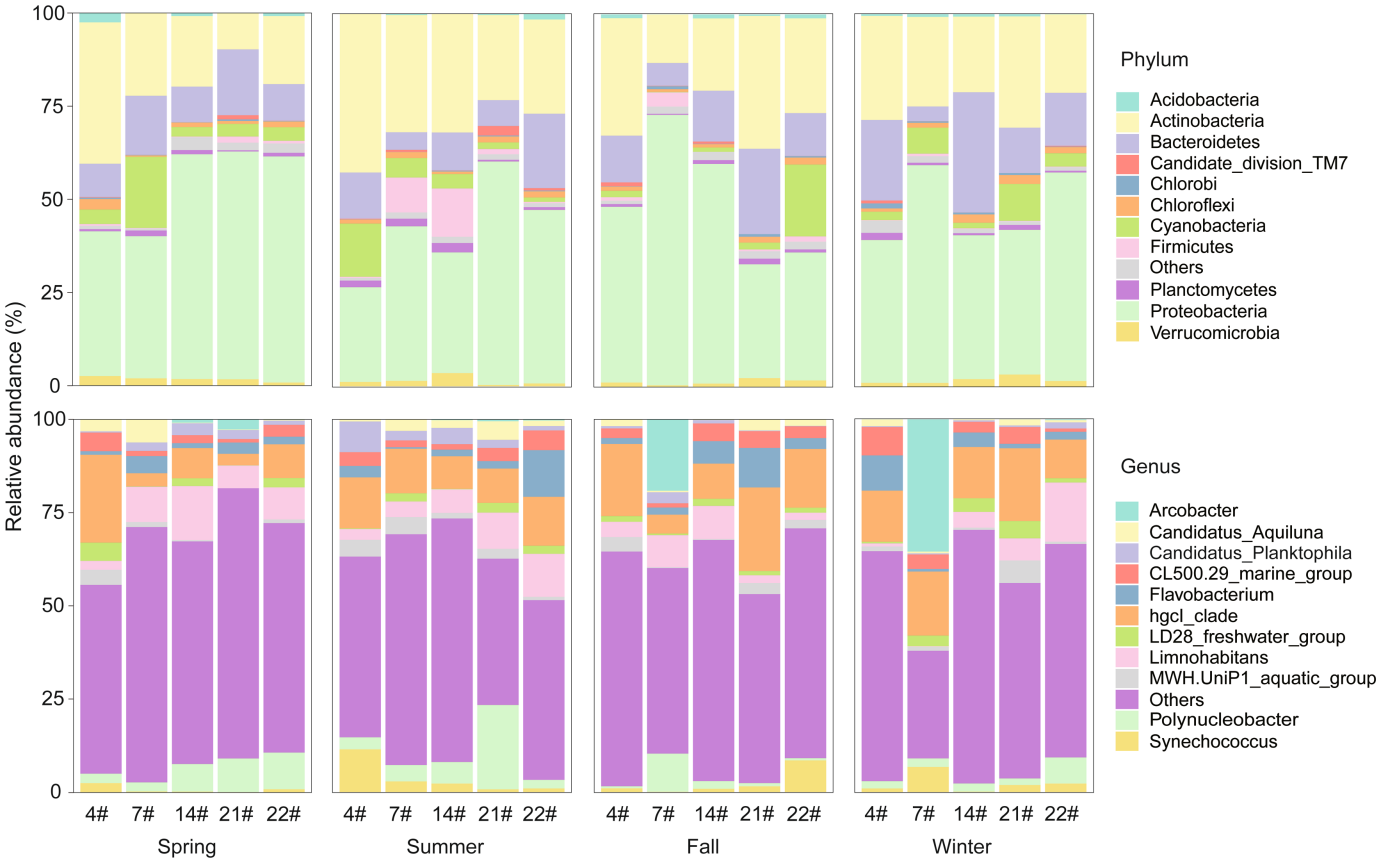
Relative abundance of bacterial taxonomy at the phylum and genus levels in Lake Bosten. The top 11 phyla/genera with relative abundance were selected to draw the percentage accumulation map, and the remaining phyla/genera were classified into others.

A total of 359 genera were identified in Lake Bosten. The major genera were *hgcI_clade* (12.9%), *Limnohabitans* (6.2%), *Polynucleobacter* (4.7%), *Flavobacterium* (3.5%), *Arcobacter* (3.3%), and *CL500-29 Marine group* (3.3%). The dominant genus, *hgcI_clade*, was present in most sites but varied in relative abundance over time and space. In spring, *Limnohabitans* and *Polynucleobacter* occupied a significant proportion, except at site 4#. The dominance of *hgcI_clade* was more consistent across sites, with less apparent variation among locations in summer. *Arcobacter* became the dominant genus at site 7# in fall and winter. These findings highlight the dynamic nature of the bacterial community composition in Lake Bosten, with variations observed across seasons and sampling sites (Figure 2).

### 3.3. Diversity of bacterial communities in Lake Bosten

**Figure 3:**
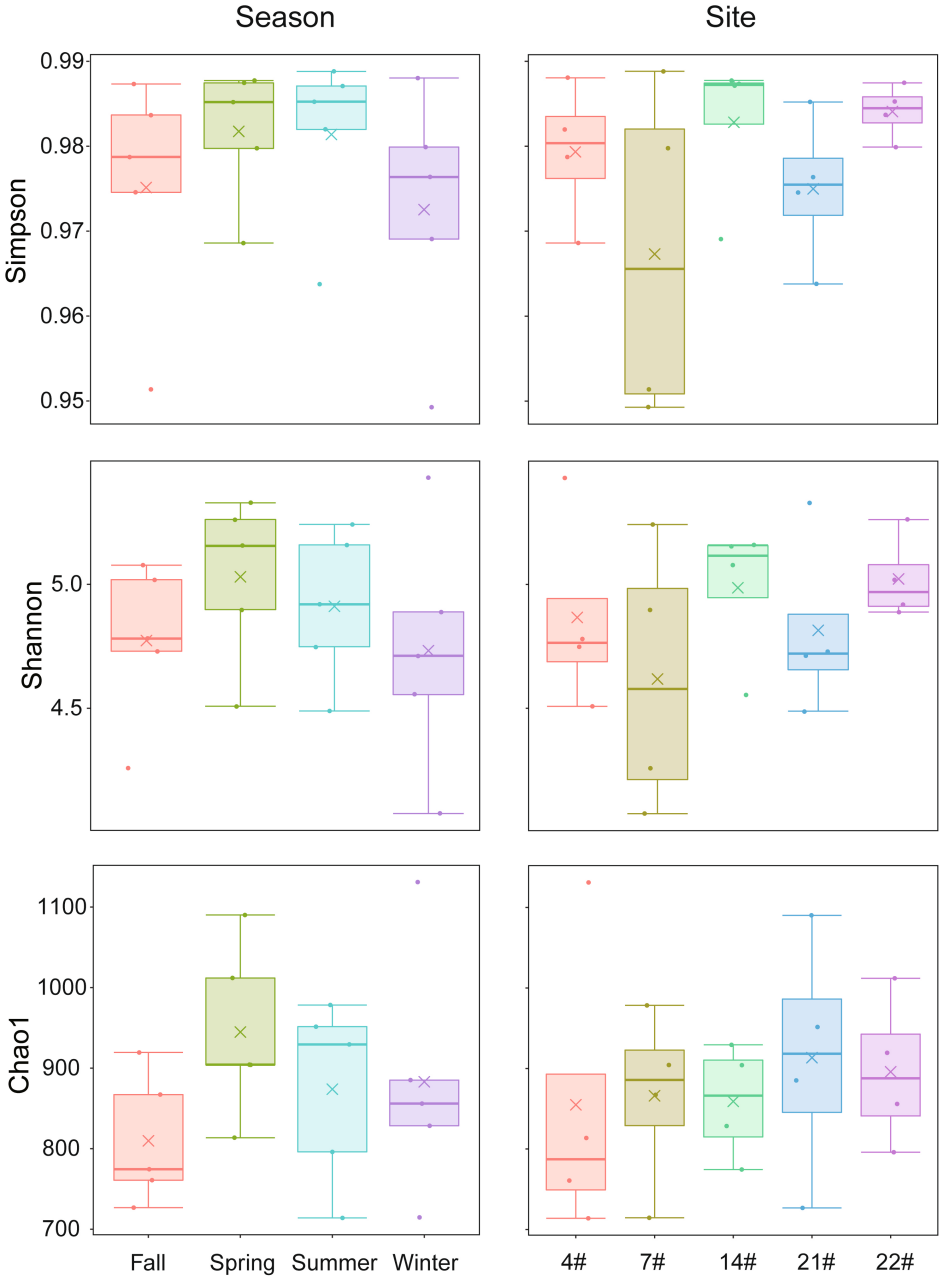
Seasonal and spatial variations of α-diversity indices in Lake Bosten. The symbol “×” in the figure represents the mean data of each group. Kruskal-Wallis test was used to analyze differences between groups.

The α-diversity of the bacterial communities in Lake Bosten is summarized in Figure 3. The Simpson and Shannon indices showed slightly higher values in spring and summer compared to fall and winter, while the Chao1 index was lowest in fall and highest in spring. No significant differences were found in the alpha diversity indices among the four seasons (*p* > 0.05), suggesting that the diversity of bacterial communities did not vary significantly throughout the year.

The Simpson and Shannon indices exhibited the highest values at site 22# and the lowest values at site 7#. The Chao1 index showed the highest value at site 21# and the lowest value at site 4#. There were no significant differences in the alpha diversity indices among the different sampling sites (*p* > 0.05), indicating that the diversity of bacterial communities did not significantly differ spatially.

**Figure 4:**
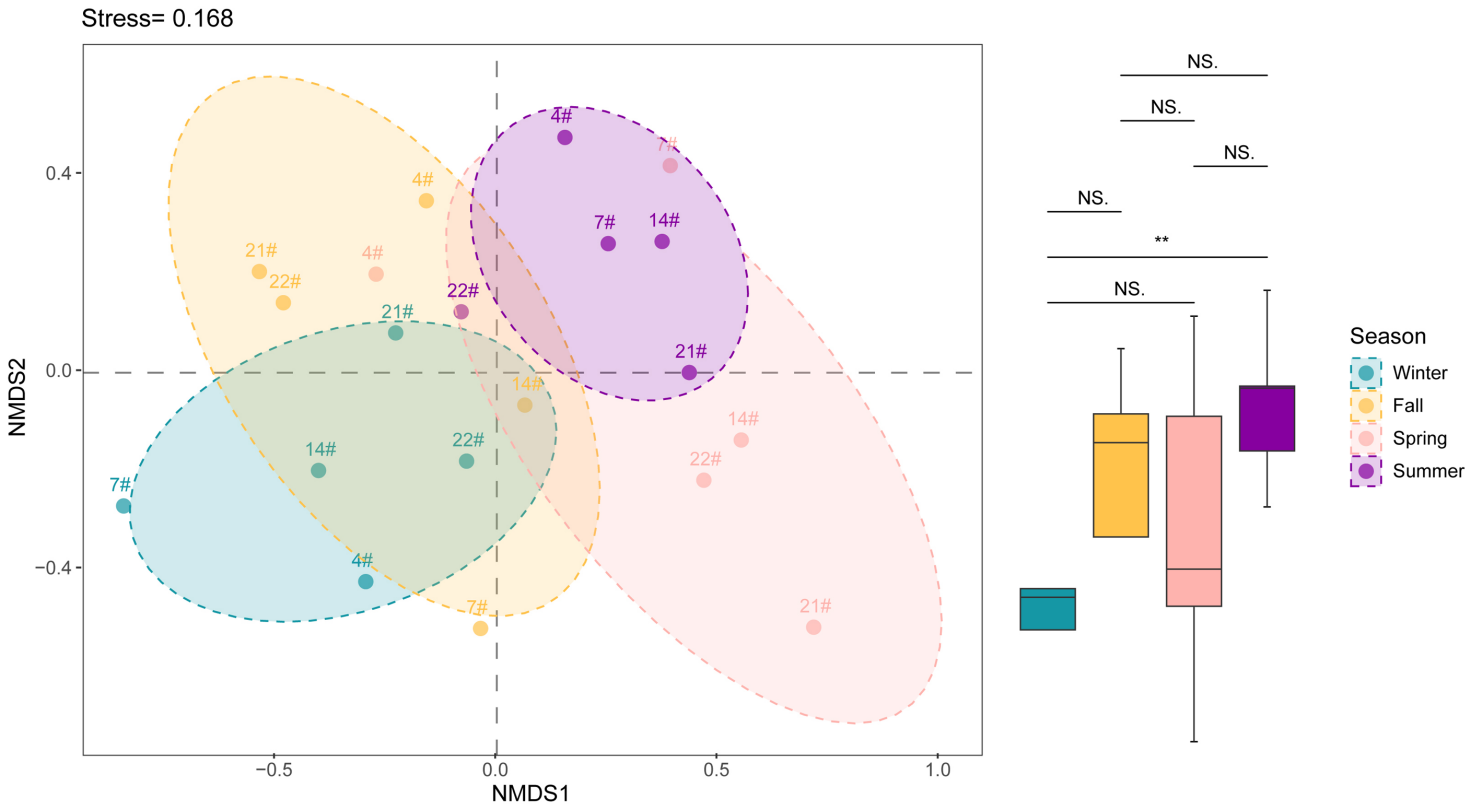
NMDS analysis showing the variation of beta diversity in Lake Bosten. ANOSIM analysis was used to estimate the differences between groups and *p* value indicated significant levels (^**^p < 0.01; NS., non-significant).

The nMDS plot (Figure 4) indicates a significant separation in the bacterial community structure between summer and winter. The separation suggests distinct microbial compositions during these seasons. The separation between summer and winter communities was found to be statistically significant (*p* < 0.05), indicating that the bacterial communities in Lake Bosten undergo seasonal shifts. The nMDS analysis did not reveal an obvious separation between different spatial locations. The bacterial community structure at the various sampling sites (spatial locations) appear to be more similar to each other compared to the temporal variations. The lack of separation between spatial locations suggests that there were no significant differences in bacterial community structure among the sampled sites, as supported by the available data (Supplementary Figure S3). These findings suggest that temporal factors, such as seasons, play a more prominent role in shaping the bacterial community structure in Lake Bosten compared to spatial factors.

### 3.4. Relationship between bacteria community and environmental factors in Lake Bosten

**Figure 5:**
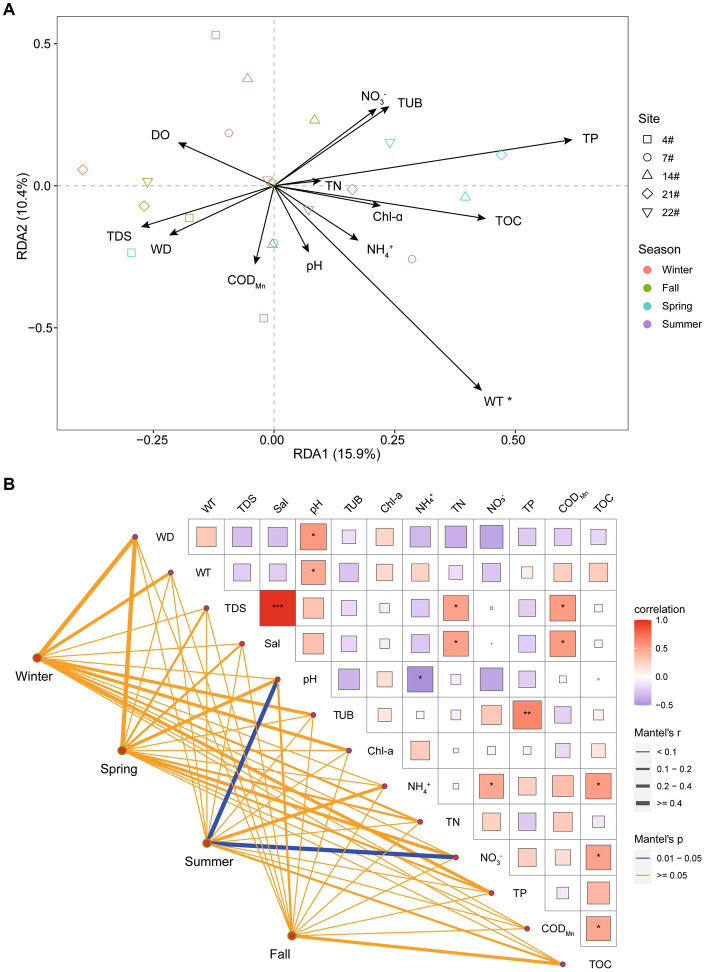
**(A)** Redundancy analysis (RDA) illustrating the relationship between bacterial communities and the environmental parameters in Lake Bosten. The collinear factors were removed by variance inflation factor analysis (VIF analysis). **(B)** Pearson’s correlation coefficients of the main 13 environmental parameters (Euclidean distance), seasons (Bray-Curtis distance), and sites (Bray-Curtis distance) of bacterial communities using Mantel permutation tests. The edge width corresponds to the correlation coefficient, and the edge color indicates statistical significance.

The results of the RDA indicated that Axis 1 and Axis 2 were significantly associated with the variation in bacterial communities in Lake Bosten, explaining 16.5% and 10.1% of the observed variation, respectively. The analysis revealed that water temperature (WT) was the most significant environmental factor (*p* < 0.05) influencing the spatial and temporal variation in bacterial community structure at the OTU level (Figure 5).

Additionally, the Mantel test analysis, which assessed the correlation between the bacterial community structure and environmental factors, suggested that different factors drove the community variation in each season. Specifically, in summer, pH and NO_3_^−^ were identified as the main driving factors shaping the bacterial community structure.

### 3.5. Co-occurrence network analysis of Lake Bosten

The co-occurrence network analysis revealed interesting patterns in the bacterial community of Lake Bosten. All co-occurrence networks in different seasons showed non-randomly structured patterns and high “small-world” properties. The network varied in terms of the number of nodes (bacterial taxa) and edges (connections between taxa) across different seasons. In spring, the network consisted of 505 nodes and 4,367 edges, while in summer, it had 430 nodes and 2,669 edges. Fall had 521 nodes and 2,874 edges, and winter had 481 nodes and 4,100 edges (Supplementary Table S2). These variations in network size indicate differences in the complexity and connectivity of the bacterial community structure throughout the year.

**Figure 6:**
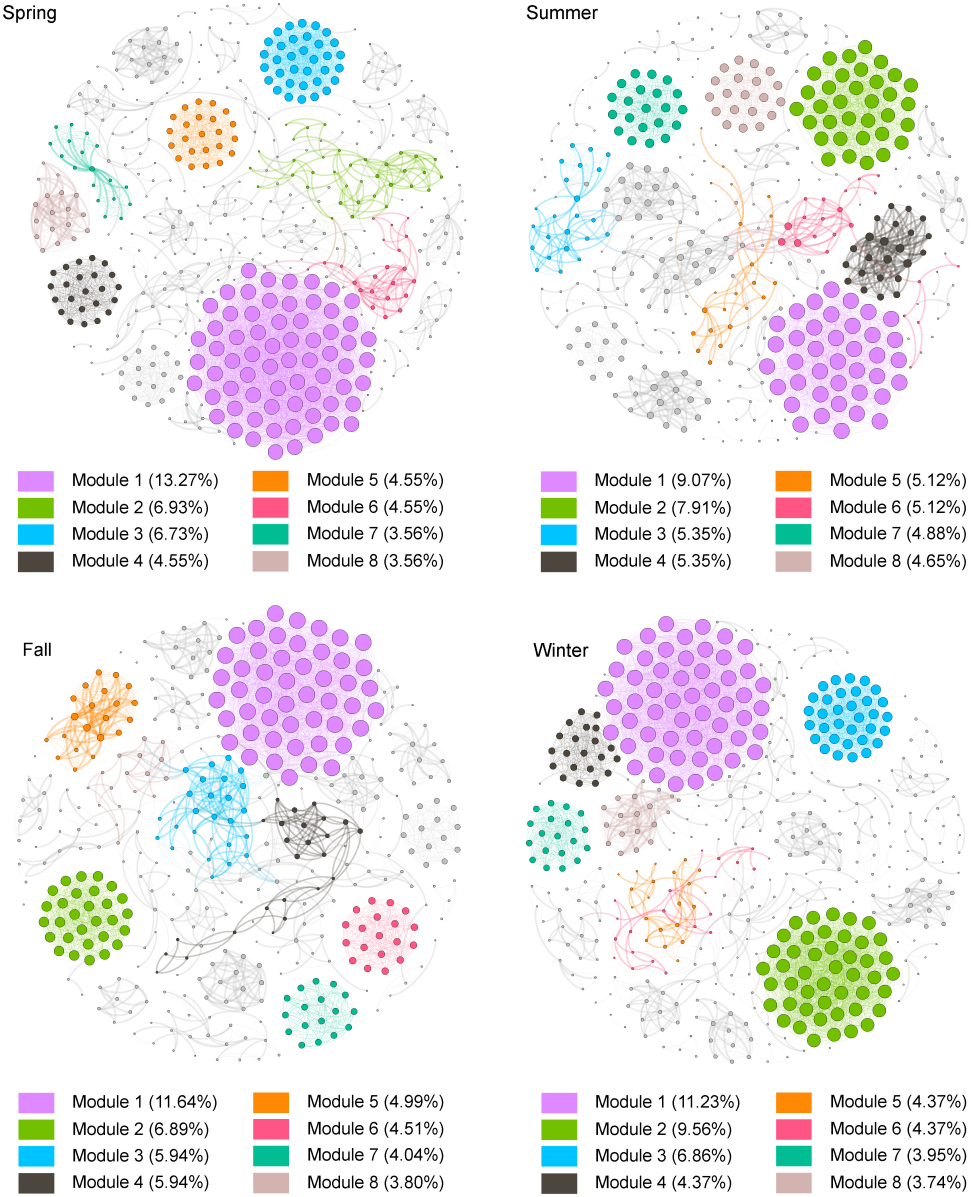
Co-occurrence networks and their topological properties for bacterial communities in Lake Bosten. OTUs were selected by their relative abundance (≥ 0.05%) among the total bacterial sequences. A connection represents a significant abs(*r*) > 0.85 (*p* < 0.01). The nodes in the networks were organized based on the number of nodes in each module, sorted from largest to smallest and filled with color. The size of the nodes in the network was determined by the value of degree, with larger nodes representing higher degrees.

Some topology parameters were displayed that the community structure of Lake Bosten exhibited a similar symbiotic pattern in spring and winter (Supplementary Table S3), suggesting some consistency in the interactions between bacterial taxa during these seasons. However, the network structure differed in summer and fall, particularly in summer, where network showed that the highest modularity and the lowest average degree, indicating a possibly less cohesive and more fragile community structure during summer(Shi et al., 2020; Shen et al., 2022) (Supplementary Table S2, Figure 6). At the phylum level, the bacterial networks remained relatively stable, primarily consisting of Proteobacteria, Bacteroidetes, Actinobacteria, and Firmicutes (Supplementary Figure S4). These phyla consistently played important roles in the bacterial community dynamics throughout the seasons.

**Figure 7:**
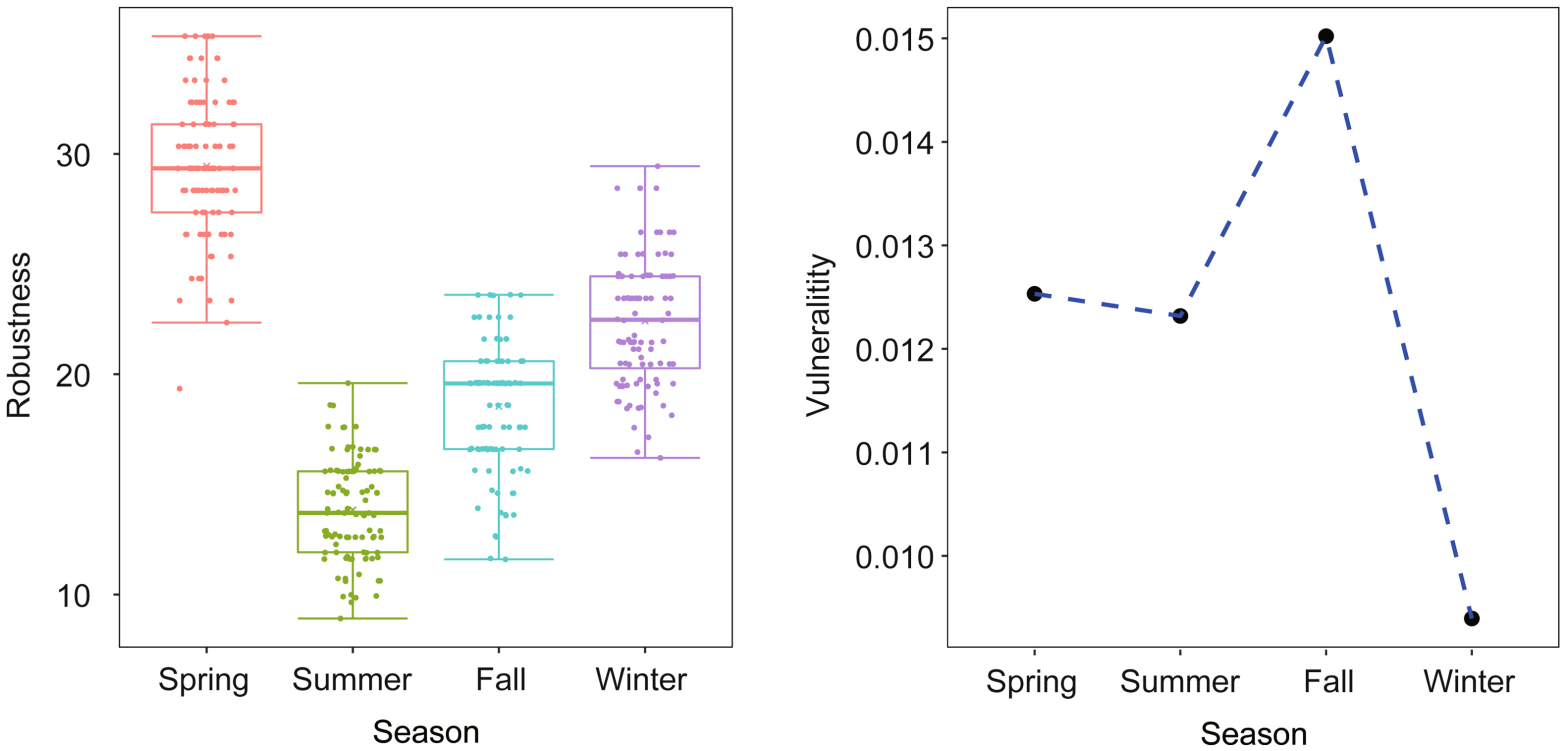
Comparison of network topological properties among networks of different seasons. Robustness is measured as the proportion of remaining taxa in a network after random node (50%) removal. Vulnerability is measured by the maximum node vulnerability in each network.

The analysis of robustness and vulnerability, which are topological properties related to network stability, revealed interesting patterns. The robustness index was higher in spring than in to the other seasons, indicating a more resilient network during that time. On the other hand, the vulnerability index was low in winter but high in fall, with spring and summer falling somewhere in between (Figure 7). These results highlight the dynamic nature of network stability across different seasons.

The identification of keystone species, which are taxa with high importance in maintaining community structure and stability, showed that all seasons except summer contained a certain number of keystone species. Spring, fall, and winter had 10 phyla and 67 genera, 10 phyla and 49 genera, and 11 phyla and 53 genera, respectively (Supplementary Table S4). These keystone species likely play crucial roles in shaping the network structure and functioning of the bacterial community in Lake Bosten.

### 3.6. Stochastic and deterministic assembly assessment

**Figure 8:**
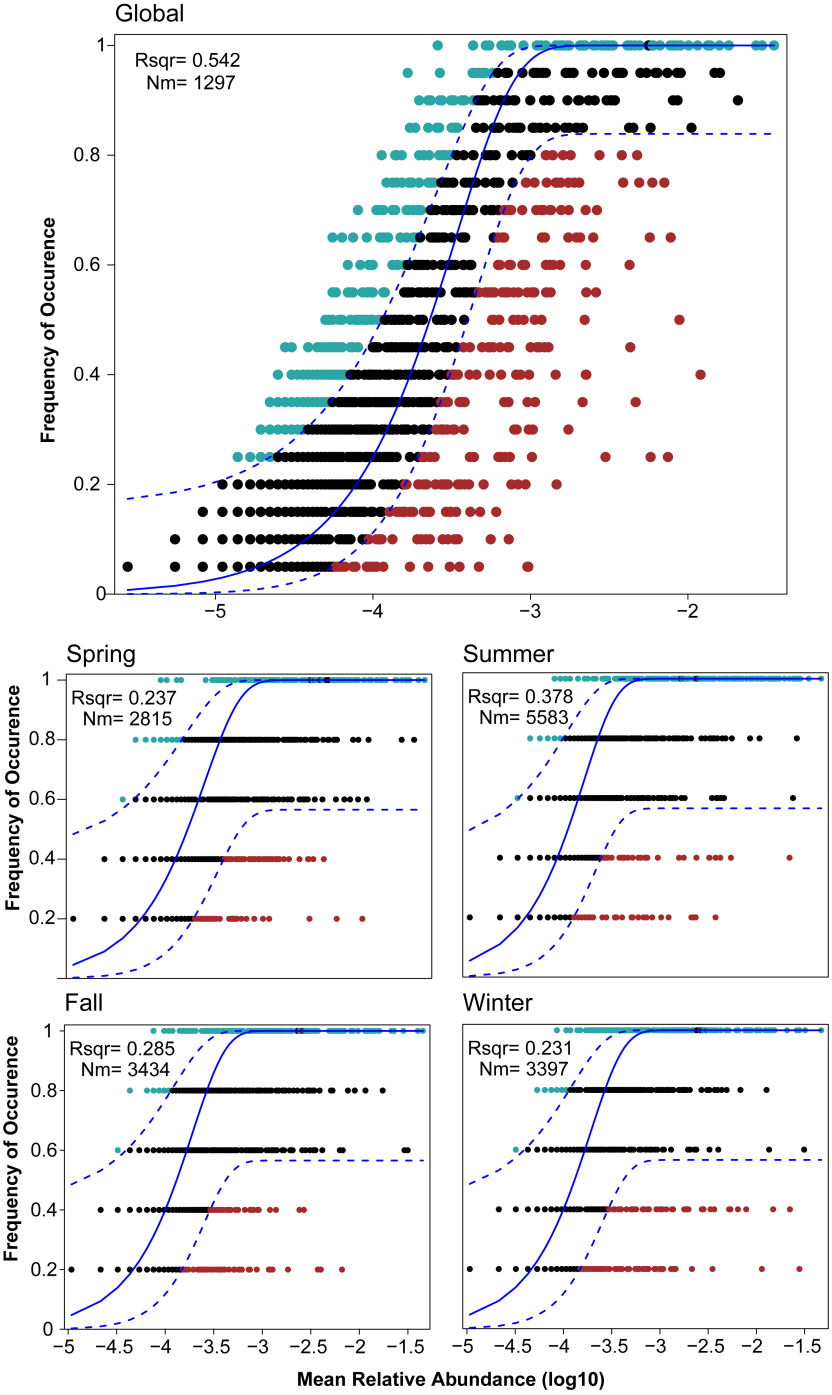
Fit of Sloan’s neutral model for analysis of bacterial community assembly globally and seasonally. The solid blue line represented the best-fitting neutral model. The dashed line represented the 95% confidence intervals (CIs) around the best-fitting neutral model. OTUs within the CIs (black points) followed the neutral process. OTUs that occur more frequently than predicted by the model were shown in red, whereas those that occur less frequently than predicted were shown in blue. *m* quantified the estimated migration rate, *N* described meta-community size, which was the total abundance of all OTUs in each sample and *r*^2^ indicated the fit to the neutral model.

The assessment of stochastic processes in shaping the bacterial community in Lake Bosten provides valuable insights into the assembly mechanisms of the community. The overall analysis indicated that stochastic processes accounted for 54.2% of the community assembly, suggesting that randomness played a dominant role in structuring the bacterial community in the lake. However, when considering seasonal differences and the succession rate of bacterial communities, the proportion of stochastic processes varied across seasons. In spring, fall, and winter, the proportion of stochastic processes was relatively small, ranging from 23.1% to 37.8% (see Figure 8). This indicates that deterministic processes, such as environmental filtering or species interactions, played a more significant role in shaping the community during these seasons. Interestingly, in summer, the proportion of stochastic processes increased by approximately 10% compared to the other seasons. This suggests that during the summer season, random factors and chance events had a more pronounced influence on the assembly of the bacterial community in Lake Bosten. These stochastic processes may include dispersal limitation, ecological drift, or random colonization events. The observed seasonal differences in the proportion of stochastic processes highlight the dynamic nature of community assembly and the varying importance of deterministic and random factors in different seasons. This implies that environmental conditions, resource availability, and biological interactions may differ throughout the year, leading to variations in the mechanisms governing bacterial community assembly.

### 3.7. Functional analysis

**Figure 9:**
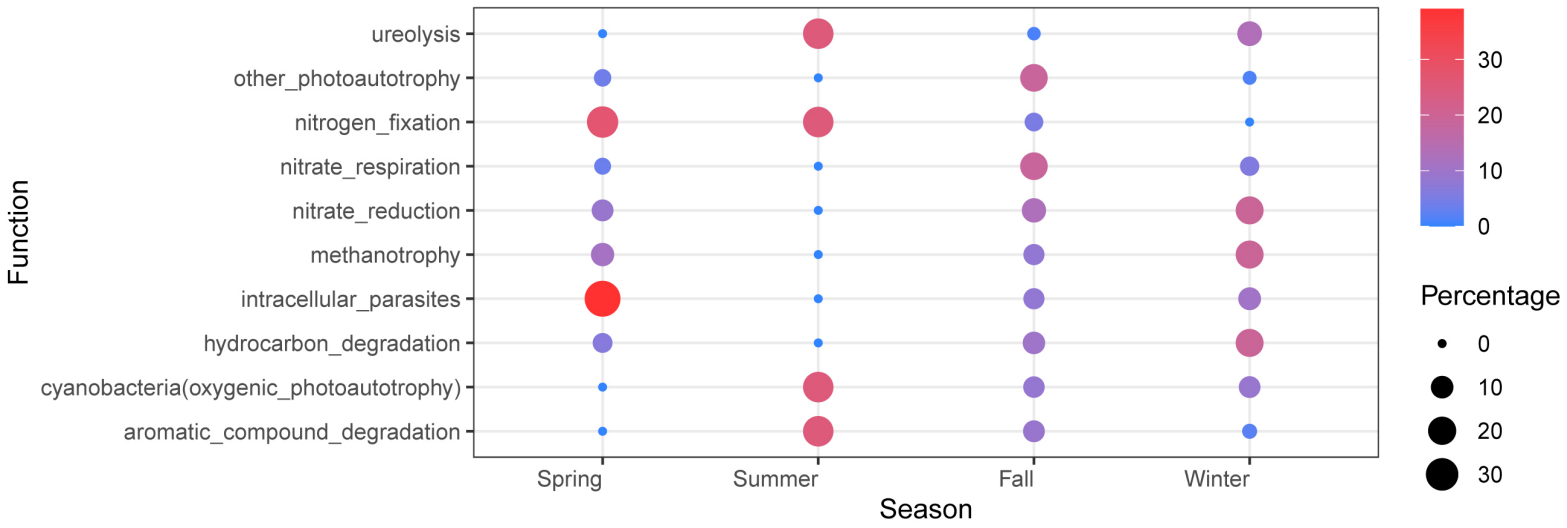
Bubble chart of OTU function in Lake Bosten. The seasonal values of each function are normalized and functions with a summer value of 1 or 0 and relative abundance in the top 20 will be retained.

The functional analysis of dominant bacteria in Lake Bosten revealed that the main functional groups were chemoheterotrophic and oxyheterotrophic bacterial communities. These functional groups are commonly associated with the utilization of organic compounds as energy sources and the consumption of oxygen in their metabolic processes (Supplementary Figure S5). Interestingly, there were no significant spatiotemporal differences observed in functional predictions, indicating that the functional composition of the bacterial community remained relatively stable across different sites and seasons. This suggests that the functional potential of the bacterial community in Lake Bosten is relatively consistent and not strongly influenced by spatial or temporal factors. However, when specifically comparing the functional differences between summer and other seasons, certain variations were observed. In summer, the functions related to photosynthesis, nitrogen fixation, and decomposition of organic matter showed higher abundance compared to other seasons. This suggests that during the summer season, there may be increased metabolic activities associated with photosynthetic bacteria, nitrogen-fixing bacteria, and decomposers, potentially driven by the availability of light, nutrients, and organic matter inputs. On the other hand, the function of nitrate respiration was found to be lowest in summer compared to other seasons. Figure 9 visually represents the differences in functional predictions between summer and other seasons, highlighting the specific functions that exhibit variation during the summer season.

## 4. Discussion

### 4.1. The main driving factors of the bacterial community structure in Lake Bosten

The bacterial community composition of Lake Bosten is characterized by the predominance of Proteobacteria, Actinobacteria, Bacteroidetes, Cyanobacteria, and Firmicutes at the phylum level. These findings align with previous studies conducted by Tang et al. (2012) and Zhang et al. (2020). Proteobacteria and Actinobacteria are commonly found in freshwater lakes worldwide (Spain et al., 2009; Tang et al., 2015; Kong et al., 2018). *Limnohabitans* and *Polynucleobacter*, both belonging to the Proteobacteria phylum, are particularly abundant in freshwater surface waters and are considered key players in freshwater ecosystems (Nuy et al., 2020). It is also one of the most studied bacterial communities (Newton et al., 2006). *HgcI_clade*, associated with Actinobacteria, has been widely detected in various freshwater environments, including lakes of different trophic states, reservoirs, rivers, and even oligosaline water (Liu et al., 2015; Aguilar et al., 2018; Camara dos Reis et al., 2019; Cruaud et al., 2019; Mohapatra et al., 2019). Firmicutes, Bacteroidetes, and Cyanobacteria are predominant phyla in the planktonic prokaryote communities of lakes, with Cyanobacteria often reflecting eutrophication status due to nitrogen and phosphorus inputs (Qin et al., 2019; Chakraborty et al., 2020), while Firmicutes and Bacteroidetes are commonly associated with the human intestinal environment (Fan and Pedersen, 2021; Zahran et al., 2021).

The seasonal WT was identified as a crucial factor driving the variation in the bacterial community structure of Lake Bosten (Figure 5A). The species diversity estimators, including Chao1 richness and Shannon indices, exhibited seasonal variations (Figure 3). Unlike previous studies on sediment bacterial communities in Lake Bosten, which indicated that WT variations weakened interspecific competition and increased the relative abundance of certain bacteria (Zhang et al., 2020), the microbial communities in the water were more influenced by WT fluctuations. Surface water, in particular, is highly susceptible to environmental factors due to its direct exposure to the external environment at the water-air interface (Pilla et al., 2021). Significant changes in atmospheric temperature can have cascading effects on plankton growth rates, lake primary productivity, and the physical and chemical environment of the lake (Lewandowska et al., 2012). Cyanobacteria, as primary producers, can stimulate the growth of heterotrophic bacteria through the release of organic matter produced via photosynthesis and respiration, consequently affecting water quality parameters such as dissolved oxygen (Zhu et al., 2013).

Furthermore, different bacterial species exhibit varying adaptations to WT. Cyanobacteria, although having an optimal growth temperature range of 25–35°C, can survive in water temperatures lower than 10°C. On the other hand, Bacteroidetes display higher growth rates at lower temperatures, making them more abundant during cold seasons such as winter (Arandia-Gorostidi et al., 2017).

Moreover, the influence of WT on the bacterial community structure is also influenced by seasonal variations in exogenous inputs. Lake Bosten receives inflows from the River Kaidu and the River Huangshui, as well as surrounding agricultural drainages (Tang et al., 2012). These inflows exhibit seasonal differences, with the River Kaidu mainly relying on glacier melt and accounting for the majority of the lake’s external inflow. In contrast, agricultural activities in the vicinity of Lake Bosten and the River Huangshui result in the discharge of high-salinity agricultural wastewater. The increase in inflow and river input during summer may explain the higher abundance of Firmicutes observed during this season. Additionally, the dominant bacterial genus at the River Kaidu estuary shifts from *hgcI_clade* to *Limnohabitans* during spring and summer, consistent with previous studies (Hu et al., 2018). The presence of *Arcobacter* dominating the estuary during fall and winter aligns with its association with animal diseases and livestock manure (Chinivasagam et al., 2007), thus reflecting the impact of agricultural practices.

### 4.2. Bacterial assembly mechanism and community stability in Lake Bosten

Based on the information provided, it appears that the community assembly of the bacterial community in Lake Bosten is influenced by both stochastic and deterministic processes, depending on the season. Overall, stochastic processes account for a significant portion (54.2%) of the community assembly, which can be attributed to the severe environmental disturbance in Lake Bosten (Guo et al., 2015; Li et al., 2021), such as extreme temperature variations and low annual planktonic microorganism numbers (Rose and Caron, 2007; Bakermans and Skidmore, 2011; Manna et al., 2019). These factors weaken interspecific competition and indirectly increase the influence of stochastic processes such as ecological drif—the bacterial abundance is only 10^5^ orders of magnitude in fall and winter (Supplementary Table S1), which is close to the critical level of ecological drift (Louca et al., 2018).

However, within each season, deterministic processes seem to play a more prominent role in shaping the bacterial community structure. The composition of the bacterial community in different seasons shows clear patterns, indicating that environmental selection and species relationships play a crucial role in community structure (Dini-Andreote et al., 2015). This observation aligns with similar studies focusing on single environmental stresses, such as lake elevation (Aguilar and Sommaruga, 2020), salinity gradient (Liu et al., 2022), geographic scale (Xiao et al., 2022), and nutrient concentration (Zeng et al., 2019).

The presence of driving factors in Lake Bosten contributes to an increase in stochastic community assembly. While randomness tends to dominate the initial phase of community succession, as community succession progresses, deterministic processes become more dominant (Dini-Andreote et al., 2015). The adaptability of microorganisms allows community succession to occur rapidly, typically within 2 weeks for certain processes (Hu et al., 2018). Therefore, from a global perspective, the community assembly mechanism may indicate that the bacterial community in Lake Bosten is in a changing and unstable state for a long time. This may have something to do with changing environmental factors (Geng et al., 2022). The driving factors—nitrate and pH—are present in Lake Bosten only in summer, indicating a certain degree of nitrogen limitation in Lake Bosten, which shows the improvement of nitrogen fixation by plankton and nitrate assimilation; and a higher pH means an increase in plankton like cyanobacteria. The network analysis indicates less cohesive and more fragile network, smaller nodes and edges, and the absence of key species in summer. This can be attributed to the variations and fluctuations of ecological effects in dominant environmental factors’ combinations, increasing the complexity and variability of interspecific relationships, weakening the status and role of key or constructive species(Banerjee et al., 2018), and making the stochastic effects of microbial communities in Bosten Lake water higher in summer.

For the other seasons, there is a hypothesis suggesting that niche assembly applies in areas with low migration rates, while diffusion assembly applies in regions with high migration rates (Loke and Chisholm, 2023). In the case of Lake Bosten, the migration rate of species entering or leaving the lake is low during spring, fall, and winter due to decreased incoming water. This observation aligns with the dominance of deterministic processes in those seasons.

## 5. Conclusion

The study conducted on Lake Bosten confirmed that the bacterial community structure exhibited spatio-temporal patterns. The major bacterial phyla identified across various sampling sites and seasons were Proteobacteria, Actinobacteria, Bacteroidetes, and Cyanobacteria. The dominant genera observed were *HgcI_clade*, *Limnohabitans*, and *Polynucleobacter*. Water temperature (WT) emerged as the most influential factor driving the spatio-temporal variations in bacterial communities. However, pH and nitrate (NO_3_^−^) levels were also identified as key factors influencing the bacterial community structure, particularly when considering only seasonal variations. The bacterial communities exhibited distinct seasonal symbiotic patterns, especially during summer. Deterministic processes primarily governed the community structure, but the role of deterministic processes decreased in summer. The functions related to photosynthesis, nitrogen fixation, and decomposition of organic matter showed higher abundance compared to other seasons.

## Data availability statement

The datasets presented in this study can be found in online repositories. The names of the repository/repositories and accession number(s) can be found at: http://bigd.big.ac.cn/gsa, CRA011126.

## Author contributions

HL: Data curation, Formal analysis, Methodology, Writing – original draft, Writing – review & editing, Visualization. JD: Resources, Writing – original draft, Data curation. ZF: Supervision, Writing – review & editing. BY: Visualization, Writing – original draft. HW: Supervision, Writing – review & editing. YH: Writing – original draft, Investigation. KS: Writing – original draft, Investigation. GG: Writing – review & editing, Conceptualization, Supervision, Validation. XT: Conceptualization, Writing – review & editing, Methodology, Resources, Supervision.

## Funding

The author(s) declare financial support was received for the research, authorship, and/or publication of this article. This work was supported by the National Key R&D Program of China (2022YFC3203902 and 2022YFC3202104), the National Natural Science Foundation of China (grant number: 41971062) and the National Natural Science Foundation of China (U2003205).

## Conflict of interest

The authors declare that the research was conducted in the absence of any commercial or financial relationships that could be construed as a potential conflict of interest.

## Publisher’s note

All claims expressed in this article are solely those of the authors and do not necessarily represent those of their affiliated organizations, or those of the publisher, the editors and the reviewers. Any product that may be evaluated in this article, or claim that may be made by its manufacturer, is not guaranteed or endorsed by the publisher.

## Footnotes

^1^https://www.r-project.org

^2^http://www.cloudtutu.com

^3^http://bigd.big.ac.cn/gsa
