# Supplementary material for: Bacterial community assembly driven by temporal succession rather than spatial heterogeneity in Lake Bosten: a large lake suffering from eutrophication and salinization
Source: Front Microbiol. 2023 Sep 20;14:1261079. doi: 10.3389/fmicb.2023.1261079 (PMC10552925; doi:10.3389/fmicb.2023.1261079)
Supplement: Supplementary file 1 [file Table_1.docx]

Table S1: Physicochemical parameters of Lake Bosten

| **Sample** | **TDS**  **mg/L** | **Sal**  **‰** | **pH** | **Turb**  **NTU** | **DO**  **mg/L** | **BA**  **ind/mL** | **Chl-a**  **μg/L** | **NH_4_^+^**  **mg/L** | **TN**  **mg/L** | **NO_3_^-^**  **mg/L** | **TP**  **mg/L** | **COD_Mn_**  **mg/L** | **Cl^-^**  **mg/L** | **SO_4_^2-^**  **mg/L** | **TOC**  **mg/L** | **SD**  **m** | **WT**  **℃** |
| --- | --- | --- | --- | --- | --- | --- | --- | --- | --- | --- | --- | --- | --- | --- | --- | --- | --- |
| 4# Winter | 1532 | 1.19 | 9.06 | 1.4 | 13.05 | 547263 | 2.7 | 0.052 | 0.961 | 0.25 | 0.012 | 5.2 | 313 | 508 | 7.6 | 3.4 | 0.76 |
| 4# Spring | 1370 | 1.08 | 9.07 | 0 | 9.43 | 572633 | 3.22 | 0.38 | 0.84 | 0.39 | 0.01 | 5.8 | 314 | 501 | 12.5 | 4.4 | 23.85 |
| 4# Summer | 1406 | 1.1 | 9.22 | 0.2 | 7.64 | 1229935 | 1.88 | 0.09 | 1.01 | 0.25 | 0 | 6.2 | 363 | 587 | 7.4 | 3.6 | 26.76 |
| 4# Fall | 1407 | 1.12 | 8.89 | 0 | 8.52 | 239201 | 2.79 | 0.09 | 1.53 | 0.33 | 0 | 5.6 | 456 | 709 | 2.5 | 3 | 14.31 |
| 7# Winter | 2068 | 1.63 | 8.94 | 7.2 | 15.55 | 489275 | 2.11 | 0.088 | 0.876 | 0.33 | 0.018 | 5.4 | 425 | 692 | 11.5 | 1.05 | 0.9 |
| 7# Spring | 1337 | 1.06 | 9.08 | 0 | 8.57 | 902440 | 2.93 | 0.37 | 0.89 | 0.35 | 0.01 | 5.4 | 313 | 499 | 16.4 | 1.2 | 26.81 |
| 7# Summer | 1853 | 1.48 | 9.1 | 0.5 | 7.99 | 1850686 | 2.84 | 0.19 | 1.53 | 0.3 | 0.018 | 6.3 | 554 | 919 | 11.1 | 1.5 | 25.25 |
| 7# Fall | 1570 | 1.25 | 8.88 | 0 | 10.14 | 1201442 | 2.98 | 0.12 | 1.4 | 0.31 | 0 | 5.9 | 337 | 600 | 5.9 | 1.2 | 9.22 |
| 14# Winter | 286 | 0.21 | 8.68 | 5.1 | 13 | 177589 | 0.82 | 0.034 | 1 | 0.9 | 0.01 | 1.3 | 28.3 | 67.6 | 1.7 | 0.7 | 0.2 |
| 14# Spring | 219 | 0.16 | 8.66 | 61.9 | 8.8 | 887943 | 4.47 | 0.38 | 0.65 | 0.56 | 0.038 | 4.6 | 11.4 | 33.2 | 24 | 0.5 | 21.2 |
| 14# Summer | 559 | 0.42 | 9 | 14.8 | 7.59 | 1187523 | 3.24 | 0.19 | 0.7 | 0.31 | 0.038 | 4 | 94.4 | 176 | 3.7 | 0.55 | 22.84 |
| 14# Fall | 1175 | 0.92 | 8.76 | 13 | 9.64 | 849888 | 8.15 | 0.17 | 1.33 | 0.29 | 0.009 | 5.1 | 429 | 819 | 4.5 | 0.6 | 9.54 |
| 21# Winter | 1792 | 1.4 | 7.56 | 7.4 | 3.92 | 414978 | 1.6 | 0.24 | 0.935 | 0.66 | 0.012 | 8.8 | 231 | 327 | 15.4 | 0.5 | 0.12 |
| 21# Spring | 1186 | 0.93 | 7.76 | 5.6 | 1.1 | 1536686 | 3.2 | 0.41 | 1.55 | 1 | 0.14 | 3.8 | 204 | 287 | 17.5 | 0.5 | 19.11 |
| 21# Summer | 1256 | 0.98 | 8.58 | 0 | 0 | 4603581 | 2.12 | 0.91 | 1.28 | 0.45 | 0 | 9.2 | 234 | 398 | 21.1 | 1.2 | 21.44 |
| 21# Fall | 1413 | 1.12 | 7.67 | 9.4 | 0.98 | 766530 | 2.84 | 0.79 | 1.79 | 0.52 | 0.016 | 8.6 | 150 | 220 | 4.3 | 0.9 | 8.53 |
| 22# Winter | 359 | 0.27 | 8.17 | 0.7 | 14.85 | 453033 | 1.5 | 0.231 | 1 | 0.25 | 0.01 | 1.6 | 30.7 | 78.6 | 1.3 | 1.6 | 4.16 |
| 22# Spring | 231 | 0.17 | 8.2 | 1.8 | 6.75 | 1699777 | 1.9 | 0.43 | 0.85 | 0.37 | 0.12 | 7.8 | 29.7 | 66.7 | 18.4 | 1.6 | 25.56 |
| 22# Summer | 271 | 0.2 | 8.75 | 1 | 5.45 | 1268491 | 1.88 | 0.14 | 0.32 | 0.11 | 0.041 | 5.3 | 9.67 | 38 | 6.5 | 2.1 | 23.49 |
| 22# Fall | 319 | 0.24 | 8.31 | 0 | 9.45 | 996671 | 3.17 | 0.22 | 0.5 | 0.19 | 0 | 2.4 | 36 | 79 | 1.6 | 2 | 10.36 |
