# Supplementary material for: Bacterial community assembly driven by temporal succession rather than spatial heterogeneity in Lake Bosten: a large lake suffering from eutrophication and salinization
Source: Front Microbiol. 2023 Sep 20;14:1261079. doi: 10.3389/fmicb.2023.1261079 (PMC10552925; doi:10.3389/fmicb.2023.1261079)
Supplement: Supplementary file 2 [file Table_2.docx]

Table S2: Co-ocurrence network topology parameters of lake Bosten in different seasons

|  | Empirical network | | | | | | | | |  | Random network | |
| --- | --- | --- | --- | --- | --- | --- | --- | --- | --- | --- | --- | --- |
|  | Nodes | Edges | |  | Modularity | avgK | avgCC | GD | σ |  | avgCC | GD |
|  |  | Positive | Negative |  |  |  |  |  |  |  |  |  |
| Winter | 481 | 4037(98.46%) | 63(1.54%) |  | 0.787 | 17.05 | 0.9914 | 0.0158 | 4420 |  | 0.035±0.001*** | 2.492±0.001*** |
| Spring | 505 | 4300(98.47%) | 67(1.53%) |  | 0.706 | 17.30 | 0.9890 | 0.0156 | 4599 |  | 0.034±0.001*** | 2.500±0.001*** |
| Summer | 430 | 2593(97.15%) | 76(2.85%) |  | 0.832 | 12.41 | 0.9574 | 0.0269 | 3290 |  | 0.030±0.002*** | 2.677±0.002*** |
| Fall | 421 | 2805(97.6%) | 69(2.4%) |  | 0.774 | 13.65 | 0.9717 | 0.0250 | 3116 |  | 0.032±0.002*** | 2.599±0.002*** |

avgK: Average degree; avgCC: Average clustering coefficient; GD: Average path distance; σ: small-word coefficient. Student's t tests were used to detect the diferences of network properties between the empirical and the random networks. ***: p < 0.001.
