# Supplementary material for: Bacterial community assembly driven by temporal succession rather than spatial heterogeneity in Lake Bosten: a large lake suffering from eutrophication and salinization
Source: Front Microbiol. 2023 Sep 20;14:1261079. doi: 10.3389/fmicb.2023.1261079 (PMC10552925; doi:10.3389/fmicb.2023.1261079)
Supplement: Supplementary file 3 [file Table_3.docx]

Table S3: p values of difference analysis of co-ocurrence network topology parameters in lake Bosten among different seasons

| **Modularity** | Winter | Spring | Summer | Fall |
| --- | --- | --- | --- | --- |
| Winter | - | 0.0001 | 0.0001 | 0.0001 |
| Spring | 0.0001 | - | 0.0001 | 0.0001 |
| Summer | 0.0001 | 0.0001 | - | 0.0001 |
| Fall | 0.0001 | 0.0001 | 0.0001 | - |

| **avgCC** | Winter | Spring | Summer | Fall |
| --- | --- | --- | --- | --- |
| Winter | - | 0.1187 | 0.0001 | 0.0001 |
| Spring | 0.1187 | - | 0.0001 | 0.0001 |
| Summer | 0.0001 | 0.0001 | - | 0.0001 |
| Fall | 0.0001 | 0.0001 | 0.0001 | - |

| **GD** | Winter | Spring | Summer | Fall |
| --- | --- | --- | --- | --- |
| Winter | - | 0.104 | 0.0001 | 0.0001 |
| Spring | 0.104 | - | 0.0001 | 0.0001 |
| Summer | 0.0001 | 0.0001 | - | 0.0001 |
| Fall | 0.0001 | 0.0001 | 0.0001 | - |

The differences of modularity, avgCC and GD among different seasons were also detected using Student's t tests.
