# Supplementary material for: Bacterial community assembly driven by temporal succession rather than spatial heterogeneity in Lake Bosten: a large lake suffering from eutrophication and salinization
Source: Front Microbiol. 2023 Sep 20;14:1261079. doi: 10.3389/fmicb.2023.1261079 (PMC10552925; doi:10.3389/fmicb.2023.1261079)
Supplement: Supplementary file 4 [file Table_4.docx]

Table S4: Keystone species and topological parameters of symbiotic networks in different seasons

Spring:

| **Id** | **name** | **phylum** | **class** | **order** | **family** | **genus** | **degree** | **Closness centrality** | **Between esscentrality** |
| --- | --- | --- | --- | --- | --- | --- | --- | --- | --- |
| n0 | 12up | Proteobacteria | Betaproteobacteria | Rhodocyclales | Rhodocyclaceae | 12up | 66 | 1 | 0 |
| n2 | Achromobacter | Proteobacteria | Betaproteobacteria | Burkholderiales | Alcaligenaceae | Achromobacter | 66 | 1 | 0 |
| n9 | Actinotalea | Actinobacteria | Actinobacteria | Micrococcales | Cellulomonadaceae | Actinotalea | 66 | 1 | 0 |
| n18 | Aquaspirillum | Proteobacteria | Betaproteobacteria | Neisseriales | Neisseriaceae | Aquaspirillum | 66 | 1 | 0 |
| n36 | Blvii28_wastewater-sludge_group | Bacteroidetes | Bacteroidia | Bacteroidales | Rikenellaceae | Blvii28_wastewater-sludge_group | 66 | 1 | 0 |
| n49 | Candidatus_Endoecteinascidia | Proteobacteria | Gammaproteobacteria | Thiotrichales | Family_Incertae_Sedis | Candidatus_Endoecteinascidia | 66 | 1 | 0 |
| n54 | Candidatus_Odyssella | Proteobacteria | Alphaproteobacteria | Rickettsiales | Family_Incertae_Sedis | Candidatus_Odyssella | 66 | 1 | 0 |
| n58 | Carnobacterium | Firmicutes | Bacilli | Lactobacillales | Carnobacteriaceae | Carnobacterium | 66 | 1 | 0 |
| n60 | Chitinophaga | Bacteroidetes | Sphingobacteriia | Sphingobacteriales | Chitinophagaceae | Chitinophaga | 66 | 1 | 0 |
| n61 | Chlorobium | Chlorobi | Chlorobia | Chlorobiales | Chlorobiaceae | Chlorobium | 66 | 1 | 0 |
| n62 | Chloroflexus | Chloroflexi | Chloroflexia | Chloroflexales | Chloroflexaceae | Chloroflexus | 66 | 1 | 0 |
| n67 | Clavibacter | Actinobacteria | Actinobacteria | Micrococcales | Microbacteriaceae | Clavibacter | 66 | 1 | 0 |
| n71 | Coxiella | Proteobacteria | Gammaproteobacteria | Legionellales | Coxiellaceae | Coxiella | 66 | 1 | 0 |
| n83 | Desulfobulbus | Proteobacteria | Deltaproteobacteria | Desulfobacterales | Desulfobulbaceae | Desulfobulbus | 66 | 1 | 0 |
| n84 | Desulfomonile | Proteobacteria | Deltaproteobacteria | Syntrophobacterales | Syntrophaceae | Desulfomonile | 66 | 1 | 0 |
| n85 | Desulforhopalus | Proteobacteria | Deltaproteobacteria | Desulfobacterales | Desulfobulbaceae | Desulforhopalus | 66 | 1 | 0 |
| n88 | Dyadobacter | Bacteroidetes | Cytophagia | Cytophagales | Cytophagaceae | Dyadobacter | 66 | 1 | 0 |
| n105 | Geitlerinema | Cyanobacteria | Cyanobacteria | SubsectionIII | FamilyI | Geitlerinema | 66 | 1 | 0 |
| n108 | Geobacter | Proteobacteria | Deltaproteobacteria | Desulfuromonadales | Geobacteraceae | Geobacter | 66 | 1 | 0 |
| n118 | Halothiobacillus | Proteobacteria | Gammaproteobacteria | Chromatiales | Halothiobacillaceae | Halothiobacillus | 66 | 1 | 0 |
| n132 | Kineosporia | Actinobacteria | Actinobacteria | Kineosporiales | Kineosporiaceae | Kineosporia | 66 | 1 | 0 |
| n136 | Lactococcus | Firmicutes | Bacilli | Lactobacillales | Streptococcaceae | Lactococcus | 66 | 1 | 0 |
| n141 | Leptolinea | Chloroflexi | Anaerolineae | Anaerolineales | Anaerolineaceae | Leptolinea | 66 | 1 | 0 |
| n146 | Loktanella | Proteobacteria | Alphaproteobacteria | Rhodobacterales | Rhodobacteraceae | Loktanella | 66 | 1 | 0 |
| n148 | Macellibacteroides | Bacteroidetes | Bacteroidia | Bacteroidales | Porphyromonadaceae | Macellibacteroides | 66 | 1 | 0 |
| n176 | norank_g_101 | Cyanobacteria | 4C0d-2 | norank_o_1 | norank_f_30 | norank_g_101 | 66 | 1 | 0 |
| n182 | norank_g_109 | Cyanobacteria | Cyanobacteria | norank_o_9 | norank_f_38 | norank_g_109 | 66 | 1 | 0 |
| n197 | norank_g_129 | Candidate_division_OP8 | norank_c_11 | norank_o_29 | norank_f_58 | norank_g_129 | 66 | 1 | 0 |
| n211 | norank_g_146 | Actinobacteria | OPB41 | norank_o_46 | norank_f_75 | norank_g_146 | 66 | 1 | 0 |
| n215 | norank_g_150 | Bacteroidetes | SB-1 | norank_o_50 | norank_f_79 | norank_g_150 | 66 | 1 | 0 |
| n222 | norank_g_16 | Proteobacteria | Gammaproteobacteria | Methylococcales | CABC2E06 | norank_g_16 | 66 | 1 | 0 |
| n225 | norank_g_163 | Bacteroidetes | vadinHA17 | norank_o_63 | norank_f_92 | norank_g_163 | 66 | 1 | 0 |
| n257 | norank_g_200 | Planctomycetes | Planctomycetacia | Planctomycetales | Planctomycetaceae | norank_g_200 | 66 | 1 | 0 |
| n259 | norank_g_204 | Proteobacteria | Gammaproteobacteria | Pseudomonadales | Pseudomonadaceae | norank_g_204 | 66 | 1 | 0 |
| n267 | norank_g_214 | Chlorobi | Chlorobia | Chlorobiales | SJA-28 | norank_g_214 | 66 | 1 | 0 |
| n282 | norank_g_23 | Chlorobi | Chlorobia | Chlorobiales | Chlorobiaceae | norank_g_23 | 66 | 1 | 0 |
| n284 | norank_g_24 | Chloroflexi | Chloroflexia | Chloroflexales | Chloroflexaceae | norank_g_24 | 66 | 1 | 0 |
| n338 | norank_g_79 | Chloroflexi | Chloroflexia | Chloroflexales | norank_f_8 | norank_g_79 | 66 | 1 | 0 |
| n347 | norank_g_89 | Chloroflexi | Dehalococcoidia | GIF9 | norank_f_18 | norank_g_89 | 66 | 1 | 0 |
| n348 | norank_g_9 | Proteobacteria | Betaproteobacteria | Burkholderiales | Alcaligenaceae | norank_g_9 | 66 | 1 | 0 |
| n351 | norank_g_93 | Elusimicrobia | Elusimicrobia | Lineage_I_Endomicrobia | norank_f_22 | norank_g_93 | 66 | 1 | 0 |
| n357 | Nubsella | Bacteroidetes | Sphingobacteriia | Sphingobacteriales | Sphingobacteriaceae | Nubsella | 66 | 1 | 0 |
| n367 | Parasegetibacter | Bacteroidetes | Sphingobacteriia | Sphingobacteriales | Chitinophagaceae | Parasegetibacter | 66 | 1 | 0 |
| n371 | Perlucidibaca | Proteobacteria | Gammaproteobacteria | Pseudomonadales | Moraxellaceae | Perlucidibaca | 66 | 1 | 0 |
| n373 | Phormidium | Cyanobacteria | Cyanobacteria | SubsectionIII | FamilyI | Phormidium | 66 | 1 | 0 |
| n389 | Proteiniclasticum | Firmicutes | Clostridia | Clostridiales | Clostridiaceae | Proteiniclasticum | 66 | 1 | 0 |
| n395 | Psychrobacter | Proteobacteria | Gammaproteobacteria | Pseudomonadales | Moraxellaceae | Psychrobacter | 66 | 1 | 0 |
| n402 | Rhodanobacter | Proteobacteria | Gammaproteobacteria | Xanthomonadales | Xanthomonadaceae | Rhodanobacter | 66 | 1 | 0 |
| n405 | Rhodomicrobium | Proteobacteria | Alphaproteobacteria | Rhizobiales | Hyphomicrobiaceae | Rhodomicrobium | 66 | 1 | 0 |
| n429 | Sphingobacterium | Bacteroidetes | Sphingobacteriia | Sphingobacteriales | Sphingobacteriaceae | Sphingobacterium | 66 | 1 | 0 |
| n438 | Sulfurimonas | Proteobacteria | Epsilonproteobacteria | Campylobacterales | Helicobacteraceae | Sulfurimonas | 66 | 1 | 0 |
| n440 | Sulfurospirillum | Proteobacteria | Epsilonproteobacteria | Campylobacterales | Campylobacteraceae | Sulfurospirillum | 66 | 1 | 0 |
| n445 | Syntrophorhabdus | Proteobacteria | Deltaproteobacteria | Order_Incertae_Sedis | Syntrophorhabdaceae | Syntrophorhabdus | 66 | 1 | 0 |
| n446 | Syntrophus | Proteobacteria | Deltaproteobacteria | Syntrophobacterales | Syntrophaceae | Syntrophus | 66 | 1 | 0 |
| n448 | Thermomonas | Proteobacteria | Gammaproteobacteria | Xanthomonadales | Xanthomonadaceae | Thermomonas | 66 | 1 | 0 |
| n450 | Thiocystis | Proteobacteria | Gammaproteobacteria | Chromatiales | Chromatiaceae | Thiocystis | 66 | 1 | 0 |
| n451 | Thiomonas | Proteobacteria | Betaproteobacteria | Burkholderiales | Family_Incertae_Sedis | Thiomonas | 66 | 1 | 0 |
| n452 | Thiothrix | Proteobacteria | Gammaproteobacteria | Thiotrichales | Thiotrichaceae | Thiothrix | 66 | 1 | 0 |
| n453 | Thiovirga | Proteobacteria | Gammaproteobacteria | Chromatiales | Halothiobacillaceae | Thiovirga | 66 | 1 | 0 |
| n454 | Tolumonas | Proteobacteria | Gammaproteobacteria | Aeromonadales | Aeromonadaceae | Tolumonas | 66 | 1 | 0 |
| n455 | Trichococcus | Firmicutes | Bacilli | Lactobacillales | Carnobacteriaceae | Trichococcus | 66 | 1 | 0 |
| n458 | uncultured_g_10 | Proteobacteria | Deltaproteobacteria | Desulfarculales | Desulfarculaceae | uncultured_g_10 | 66 | 1 | 0 |
| n463 | uncultured_g_15_1 | Bacteroidetes | Flavobacteria | Flavobacteriales | Flavobacteriaceae | uncultured_g_15_1 | 66 | 1 | 0 |
| n466 | uncultured_g_17 | Proteobacteria | Gammaproteobacteria | Chromatiales | Halothiobacillaceae | uncultured_g_17 | 66 | 1 | 0 |
| n467 | uncultured_g_18 | Proteobacteria | Epsilonproteobacteria | Campylobacterales | Helicobacteraceae | uncultured_g_18 | 66 | 1 | 0 |
| n487 | uncultured_g_44 | Proteobacteria | Deltaproteobacteria | Syntrophobacterales | Syntrophobacteraceae | uncultured_g_44 | 66 | 1 | 0 |
| n493 | uncultured_g_7 | Firmicutes | Clostridia | Clostridiales | Christensenellaceae | uncultured_g_7 | 66 | 1 | 0 |

Summer: None

Fall:

| **Id** | **name** | **phylum** | **class** | **order** | **family** | **genus** | **degree** | **Closness centrality** | **Betweenness centrality** |
| --- | --- | --- | --- | --- | --- | --- | --- | --- | --- |
| n2 | Acidaminobacter | Firmicutes | Clostridia | Clostridiales | Family_XII_Incertae_Sedis | Acidaminobacter | 48 | 1 | 0 |
| n11 | Aquaspirillum | Proteobacteria | Betaproteobacteria | Neisseriales | Neisseriaceae | Aquaspirillum | 48 | 1 | 0 |
| n45 | Candidatus_Odyssella | Proteobacteria | Alphaproteobacteria | Rickettsiales | Family_Incertae_Sedis | Candidatus_Odyssella | 48 | 1 | 0 |
| n53 | Chlorobium | Chlorobi | Chlorobia | Chlorobiales | Chlorobiaceae | Chlorobium | 48 | 1 | 0 |
| n60 | Clostridium | Firmicutes | Clostridia | Clostridiales | Clostridiaceae | Clostridium | 48 | 1 | 0 |
| n70 | Desulfobulbus | Proteobacteria | Deltaproteobacteria | Desulfobacterales | Desulfobulbaceae | Desulfobulbus | 48 | 1 | 0 |
| n71 | Desulfocapsa | Proteobacteria | Deltaproteobacteria | Desulfobacterales | Desulfobulbaceae | Desulfocapsa | 48 | 1 | 0 |
| n72 | Desulfomonile | Proteobacteria | Deltaproteobacteria | Syntrophobacterales | Syntrophaceae | Desulfomonile | 48 | 1 | 0 |
| n81 | Erysipelothrix | Firmicutes | Erysipelotrichia | Erysipelotrichales | Erysipelotrichaceae | Erysipelothrix | 48 | 1 | 0 |
| n91 | Fusibacter | Firmicutes | Clostridia | Clostridiales | Family_XII_Incertae_Sedis | Fusibacter | 48 | 1 | 0 |
| n93 | Geobacter | Proteobacteria | Deltaproteobacteria | Desulfuromonadales | Geobacteraceae | Geobacter | 48 | 1 | 0 |
| n98 | Halothiobacillus | Proteobacteria | Gammaproteobacteria | Chromatiales | Halothiobacillaceae | Halothiobacillus | 48 | 1 | 0 |
| n110 | Iodobacter | Proteobacteria | Betaproteobacteria | Neisseriales | Neisseriaceae | Iodobacter | 48 | 1 | 0 |
| n112 | Janthinobacterium | Proteobacteria | Betaproteobacteria | Burkholderiales | Oxalobacteraceae | Janthinobacterium | 48 | 1 | 0 |
| n122 | Macellibacteroides | Bacteroidetes | Bacteroidia | Bacteroidales | Porphyromonadaceae | Macellibacteroides | 48 | 1 | 0 |
| n146 | norank_g_101 | Cyanobacteria | 4C0d-2 | norank_o_1 | norank_f_30 | norank_g_101 | 48 | 1 | 0 |
| n171 | norank_g_140 | SM2F11 | norank_c_22 | norank_o_40 | norank_f_69 | norank_g_140 | 48 | 1 | 0 |
| n177 | norank_g_146 | Actinobacteria | OPB41 | norank_o_46 | norank_f_75 | norank_g_146 | 48 | 1 | 0 |
| n180 | norank_g_151 | Bacteroidetes | SB-5 | norank_o_51 | norank_f_80 | norank_g_151 | 48 | 1 | 0 |
| n187 | norank_g_163 | Bacteroidetes | vadinHA17 | norank_o_63 | norank_f_92 | norank_g_163 | 48 | 1 | 0 |
| n194 | norank_g_17 | Chloroflexi | Caldilineae | Caldilineales | Caldilineaceae | norank_g_17 | 48 | 1 | 0 |
| n215 | norank_g_20 | Proteobacteria | Gammaproteobacteria | Thiotrichales | CHAB-XI-27 | norank_g_20 | 48 | 1 | 0 |
| n222 | norank_g_207 | Proteobacteria | Alphaproteobacteria | Rhodospirillales | Rhodospirillaceae | norank_g_207 | 48 | 1 | 0 |
| n225 | norank_g_210 | Bacteroidetes | Bacteroidia | Bacteroidales | S24-7 | norank_g_210 | 48 | 1 | 0 |
| n234 | norank_g_223 | Chloroflexi | Dehalococcoidia | Dehalococcoidales | uncultured_f_3 | norank_g_223 | 48 | 1 | 0 |
| n247 | norank_g_3 | Proteobacteria | Alphaproteobacteria | Sphingomonadales | 7B-8 | norank_g_3 | 48 | 1 | 0 |
| n277 | norank_g_71 | Proteobacteria | Betaproteobacteria | Neisseriales | Neisseriaceae | norank_g_71 | 48 | 1 | 0 |
| n278 | norank_g_72 | Proteobacteria | Deltaproteobacteria | 43F-1404R | norank_f_1 | norank_g_72 | 48 | 1 | 0 |
| n287 | norank_g_89 | Chloroflexi | Dehalococcoidia | GIF9 | norank_f_18 | norank_g_89 | 48 | 1 | 0 |
| n301 | Paludibacter | Bacteroidetes | Bacteroidia | Bacteroidales | Porphyromonadaceae | Paludibacter | 48 | 1 | 0 |
| n327 | Psychrobacter | Proteobacteria | Gammaproteobacteria | Pseudomonadales | Moraxellaceae | Psychrobacter | 48 | 1 | 0 |
| n343 | Ruminococcus | Firmicutes | Clostridia | Clostridiales | Ruminococcaceae | Ruminococcus | 48 | 1 | 0 |
| n349 | Sideroxydans | Proteobacteria | Betaproteobacteria | Nitrosomonadales | Gallionellaceae | Sideroxydans | 48 | 1 | 0 |
| n364 | Sulfurimonas | Proteobacteria | Epsilonproteobacteria | Campylobacterales | Helicobacteraceae | Sulfurimonas | 48 | 1 | 0 |
| n366 | Sulfurospirillum | Proteobacteria | Epsilonproteobacteria | Campylobacterales | Campylobacteraceae | Sulfurospirillum | 48 | 1 | 0 |
| n367 | Sulfurovum | Proteobacteria | Epsilonproteobacteria | Campylobacterales | Helicobacteraceae | Sulfurovum | 48 | 1 | 0 |
| n370 | Syntrophus | Proteobacteria | Deltaproteobacteria | Syntrophobacterales | Syntrophaceae | Syntrophus | 48 | 1 | 0 |
| n372 | Terrimonas | Bacteroidetes | Sphingobacteriia | Sphingobacteriales | Chitinophagaceae | Terrimonas | 48 | 1 | 0 |
| n376 | Thiocystis | Proteobacteria | Gammaproteobacteria | Chromatiales | Chromatiaceae | Thiocystis | 48 | 1 | 0 |
| n377 | Thiomicrospira | Proteobacteria | Gammaproteobacteria | Thiotrichales | Piscirickettsiaceae | Thiomicrospira | 48 | 1 | 0 |
| n379 | Thiovirga | Proteobacteria | Gammaproteobacteria | Chromatiales | Halothiobacillaceae | Thiovirga | 48 | 1 | 0 |
| n380 | Tolumonas | Proteobacteria | Gammaproteobacteria | Aeromonadales | Aeromonadaceae | Tolumonas | 48 | 1 | 0 |
| n385 | uncultured_g_15_1 | Bacteroidetes | Flavobacteria | Flavobacteriales | Flavobacteriaceae | uncultured_g_15_1 | 48 | 1 | 0 |
| n387 | uncultured_g_17 | Proteobacteria | Gammaproteobacteria | Chromatiales | Halothiobacillaceae | uncultured_g_17 | 48 | 1 | 0 |
| n388 | uncultured_g_18 | Proteobacteria | Epsilonproteobacteria | Campylobacterales | Helicobacteraceae | uncultured_g_18 | 48 | 1 | 0 |
| n390 | uncultured_g_20 | Proteobacteria | Betaproteobacteria | Hydrogenophilales | Hydrogenophilaceae | uncultured_g_20 | 48 | 1 | 0 |
| n392 | uncultured_g_22 | Firmicutes | Clostridia | Clostridiales | Lachnospiraceae | uncultured_g_22 | 48 | 1 | 0 |
| n393 | uncultured_g_24 | Fusobacteria | Fusobacteriia | Fusobacteriales | Leptotrichiaceae | uncultured_g_24 | 48 | 1 | 0 |
| n407 | uncultured_g_44 | Proteobacteria | Deltaproteobacteria | Syntrophobacterales | Syntrophobacteraceae | uncultured_g_44 | 48 | 1 | 0 |

Winter:

| **Id** | **name** | **phylum** | **class** | **order** | **family** | **genus** | **degree** | **Closness centrality** | **Betweenness centrality** |
| --- | --- | --- | --- | --- | --- | --- | --- | --- | --- |
| n7 | Actibacter | Bacteroidetes | Flavobacteria | Flavobacteriales | Flavobacteriaceae | Actibacter | 53 | 1 | 0 |
| n11 | Alkanindiges | Proteobacteria | Gammaproteobacteria | Pseudomonadales | Moraxellaceae | Alkanindiges | 53 | 1 | 0 |
| n24 | Aureispira | Bacteroidetes | Sphingobacteriia | Sphingobacteriales | Saprospiraceae | Aureispira | 53 | 1 | 0 |
| n28 | Bacteriovorax | Proteobacteria | Deltaproteobacteria | Bdellovibrionales | Bacteriovoracaceae | Bacteriovorax | 53 | 1 | 0 |
| n37 | Bryobacter | Acidobacteria | Acidobacteria | Subgroup_3 | Family_Incertae_Sedis | Bryobacter | 53 | 1 | 0 |
| n39 | Candidatus_Anadelfobacter | Proteobacteria | Alphaproteobacteria | Rickettsiales | Family_Incertae_Sedis | Candidatus_Anadelfobacter | 53 | 1 | 0 |
| n49 | Candidatus_Nitrotoga | Proteobacteria | Betaproteobacteria | Nitrosomonadales | Gallionellaceae | Candidatus_Nitrotoga | 53 | 1 | 0 |
| n55 | Cellvibrio | Proteobacteria | Gammaproteobacteria | Pseudomonadales | Pseudomonadaceae | Cellvibrio | 53 | 1 | 0 |
| n58 | Chryseobacterium | Bacteroidetes | Flavobacteria | Flavobacteriales | Flavobacteriaceae | Chryseobacterium | 53 | 1 | 0 |
| n63 | Clavibacter | Actinobacteria | Actinobacteria | Micrococcales | Microbacteriaceae | Clavibacter | 53 | 1 | 0 |
| n69 | Crocinitomix | Bacteroidetes | Flavobacteria | Flavobacteriales | Cryomorphaceae | Crocinitomix | 53 | 1 | 0 |
| n75 | Desulfobacca | Proteobacteria | Deltaproteobacteria | Syntrophobacterales | Syntrophaceae | Desulfobacca | 53 | 1 | 0 |
| n96 | Gaiella | Actinobacteria | Thermoleophilia | Gaiellales | Gaiellaceae | Gaiella | 53 | 1 | 0 |
| n111 | Hymenobacter | Bacteroidetes | Cytophagia | Cytophagales | Cytophagaceae | Hymenobacter | 53 | 1 | 0 |
| n112 | Iamia | Actinobacteria | Acidimicrobiia | Acidimicrobiales | Iamiaceae | Iamia | 53 | 1 | 0 |
| n113 | Incertae_Sedis_1 | Firmicutes | Clostridia | Clostridiales | Family_XIII_Incertae_Sedis | Incertae_Sedis_1 | 53 | 1 | 0 |
| n114 | Inhella | Proteobacteria | Betaproteobacteria | Burkholderiales | Comamonadaceae | Inhella | 53 | 1 | 0 |
| n116 | Janibacter | Actinobacteria | Actinobacteria | Micrococcales | Intrasporangiaceae | Janibacter | 53 | 1 | 0 |
| n131 | Lutibacter | Bacteroidetes | Flavobacteria | Flavobacteriales | Flavobacteriaceae | Lutibacter | 53 | 1 | 0 |
| n136 | Megamonas | Firmicutes | Negativicutes | Selenomonadales | Veillonellaceae | Megamonas | 53 | 1 | 0 |
| n137 | Meganema | Proteobacteria | Alphaproteobacteria | Rhizobiales | Methylobacteriaceae | Meganema | 53 | 1 | 0 |
| n139 | Methylobacter | Proteobacteria | Gammaproteobacteria | Methylococcales | Methylococcaceae | Methylobacter | 53 | 1 | 0 |
| n162 | norank_g_104 | Bacteroidetes | BD2-2 | norank_o_4 | norank_f_33 | norank_g_104 | 53 | 1 | 0 |
| n173 | norank_g_12 | Proteobacteria | Deltaproteobacteria | Bdellovibrionales | Bacteriovoracaceae | norank_g_12 | 53 | 1 | 0 |
| n178 | norank_g_124 | BHI80-139 | norank_c_6 | norank_o_24 | norank_f_53 | norank_g_124 | 53 | 1 | 0 |
| n192 | norank_g_14 | Chlorobi | Ignavibacteria | Ignavibacteriales | BSV26 | norank_g_14 | 53 | 1 | 0 |
| n204 | norank_g_156 | Bacteroidetes | SM1A07 | norank_o_56 | norank_f_85 | norank_g_156 | 53 | 1 | 0 |
| n218 | norank_g_174 | Planctomycetes | Phycisphaerae | S15A-MN16 | norank_f_103 | norank_g_174 | 53 | 1 | 0 |
| n223 | norank_g_178 | Acidobacteria | Acidobacteria | Subgroup_17 | norank_f_107 | norank_g_178 | 53 | 1 | 0 |
| n224 | norank_g_18 | Proteobacteria | Alphaproteobacteria | Caulobacterales | Caulobacteraceae | norank_g_18 | 53 | 1 | 0 |
| n229 | norank_g_184 | Acidobacteria | Holophagae | Subgroup_7 | norank_f_113 | norank_g_184 | 53 | 1 | 0 |
| n236 | norank_g_194 | Actinobacteria | Acidimicrobiia | Acidimicrobiales | OCS155_marine_group | norank_g_194 | 53 | 1 | 0 |
| n247 | norank_g_208 | Proteobacteria | Alphaproteobacteria | Rickettsiales | Rickettsiaceae | norank_g_208 | 53 | 1 | 0 |
| n251 | norank_g_212 | Nitrospirae | Nitrospira | Nitrospirales | Sh765B-TzT-35 | norank_g_212 | 53 | 1 | 0 |
| n302 | norank_g_64 | Proteobacteria | Deltaproteobacteria | Desulfuromonadales | M20-Pitesti | norank_g_64 | 53 | 1 | 0 |
| n306 | norank_g_68 | Proteobacteria | Deltaproteobacteria | Myxococcales | mle1-27 | norank_g_68 | 53 | 1 | 0 |
| n309 | norank_g_70 | Proteobacteria | Gammaproteobacteria | Pseudomonadales | Moraxellaceae | norank_g_70 | 53 | 1 | 0 |
| n311 | norank_g_74 | Gemmatimonadetes | Gemmatimonadetes | AT425-gubC11 _terrestrial_group | norank_f_3 | norank_g_74 | 53 | 1 | 0 |
| n312 | norank_g_75 | Acidobacteria | Acidobacteria | AT-s3-28 | norank_f_4 | norank_g_75 | 53 | 1 | 0 |
| n328 | norank_g_99 | Proteobacteria | Deltaproteobacteria | Myxococcales | norank_f_28 | norank_g_99 | 53 | 1 | 0 |
| n370 | Psychrobacter | Proteobacteria | Gammaproteobacteria | Pseudomonadales | Moraxellaceae | Psychrobacter | 53 | 1 | 0 |
| n393 | Saprospira | Bacteroidetes | Sphingobacteriia | Sphingobacteriales | Saprospiraceae | Saprospira | 53 | 1 | 0 |
| n394 | Schlesneria | Planctomycetes | Planctomycetacia | Planctomycetales | Planctomycetaceae | Schlesneria | 53 | 1 | 0 |
| n401 | Solimonas | Proteobacteria | Gammaproteobacteria | Xanthomonadales | Solimonadaceae | Solimonas | 53 | 1 | 0 |
| n408 | Sporichthya | Actinobacteria | Actinobacteria | Frankiales | Sporichthyaceae | Sporichthya | 53 | 1 | 0 |
| n422 | Terrimonas | Bacteroidetes | Sphingobacteriia | Sphingobacteriales | Chitinophagaceae | Terrimonas | 53 | 1 | 0 |
| n433 | uncultured_g_1_1 | Actinobacteria | Acidimicrobiia | Acidimicrobiales | Acidimicrobiaceae | uncultured_g_1_1 | 53 | 1 | 0 |
| n438 | uncultured_g_15 | Fibrobacteres | Fibrobacteria | Fibrobacterales | Fibrobacteraceae | uncultured_g_15 | 53 | 1 | 0 |
| n450 | uncultured_g_26 | Proteobacteria | Gammaproteobacteria | Pseudomonadales | Moraxellaceae | uncultured_g_26 | 53 | 1 | 0 |
| n451 | uncultured_g_27 | Proteobacteria | Betaproteobacteria | Neisseriales | Neisseriaceae | uncultured_g_27 | 53 | 1 | 0 |
| n456 | uncultured_g_34 | Proteobacteria | Betaproteobacteria | Rhodocyclales | Rhodocyclaceae | uncultured_g_34 | 53 | 1 | 0 |
| n473 | Vibrio | Proteobacteria | Gammaproteobacteria | Vibrionales | Vibrionaceae | Vibrio | 53 | 1 | 0 |
| n475 | Woodsholea | Proteobacteria | Alphaproteobacteria | Caulobacterales | Hyphomonadaceae | Woodsholea | 53 | 1 | 0 |
| n479 | Zavarzinella | Planctomycetes | Planctomycetacia | Planctomycetales | Planctomycetaceae | Zavarzinella | 53 | 1 | 0 |
