# Supplementary figures and images for: Bacterial community assembly driven by temporal succession rather than spatial heterogeneity in Lake Bosten: a large lake suffering from eutrophication and salinization
Source: Front Microbiol. 2023 Sep 20;14:1261079. doi: 10.3389/fmicb.2023.1261079 (PMC10552925; doi:10.3389/fmicb.2023.1261079)

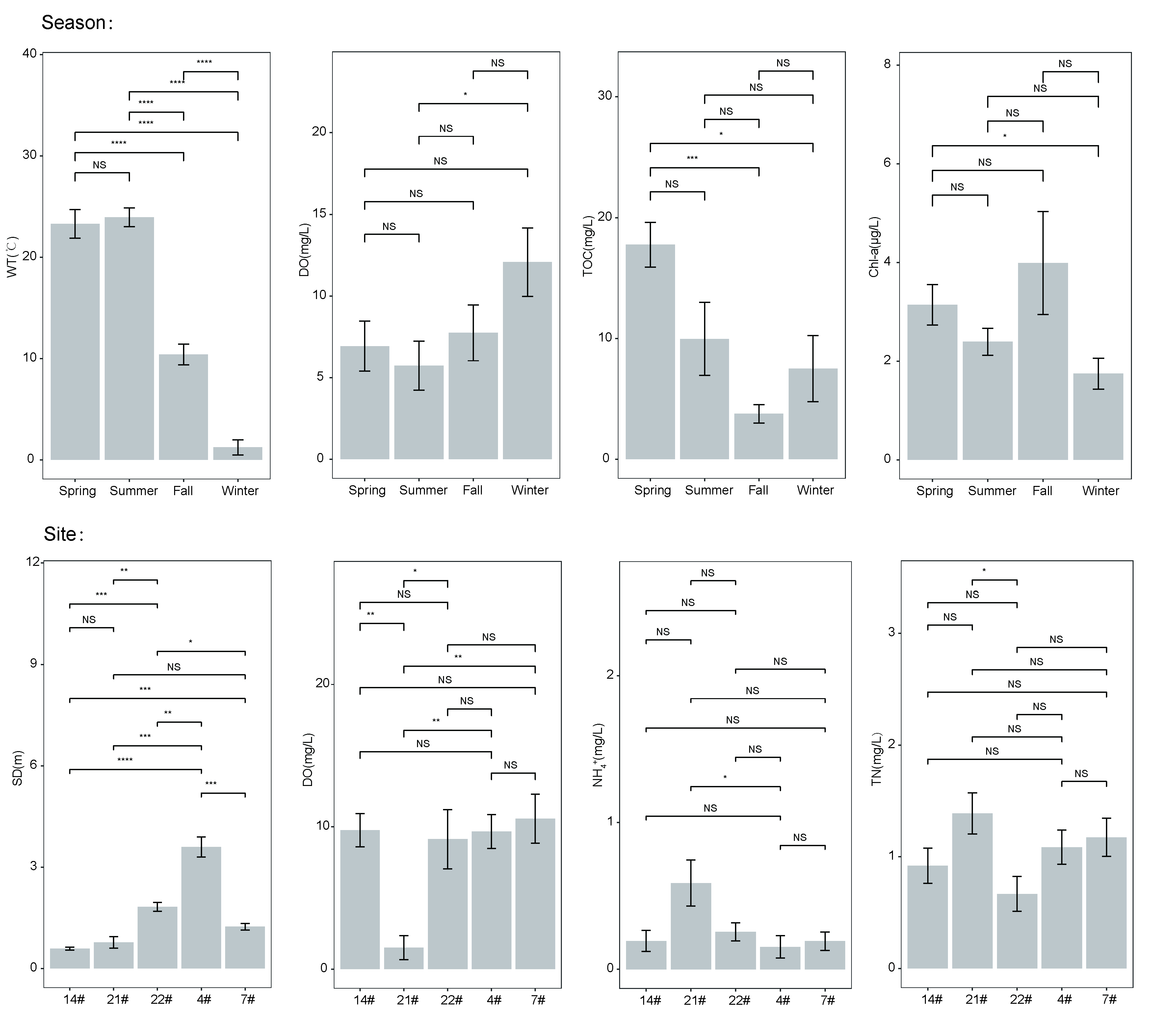

Supplement: Supplementary file 5 [file Image_1.jpeg]

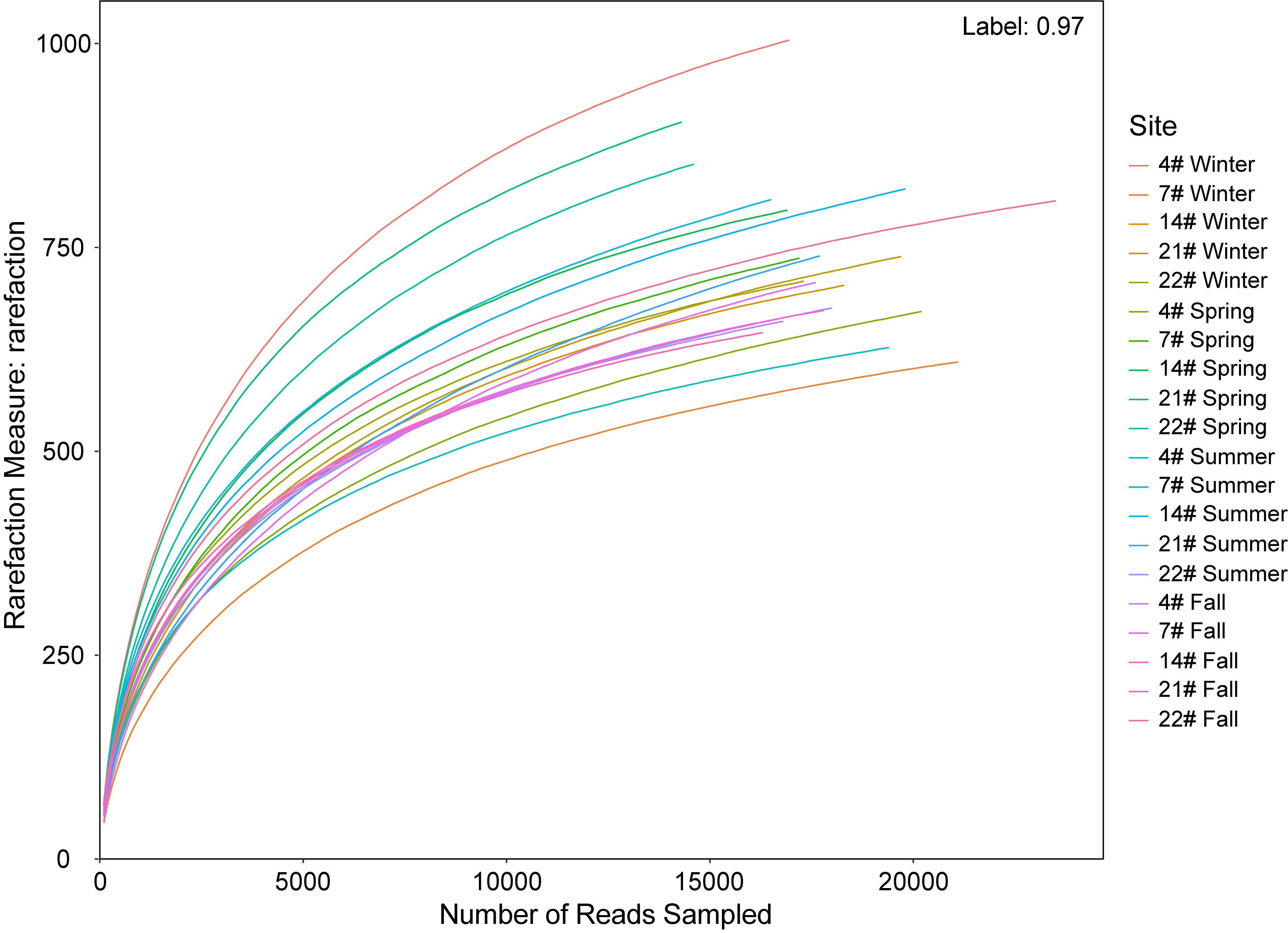

Supplement: Supplementary file 6 [file Image_2.jpeg]

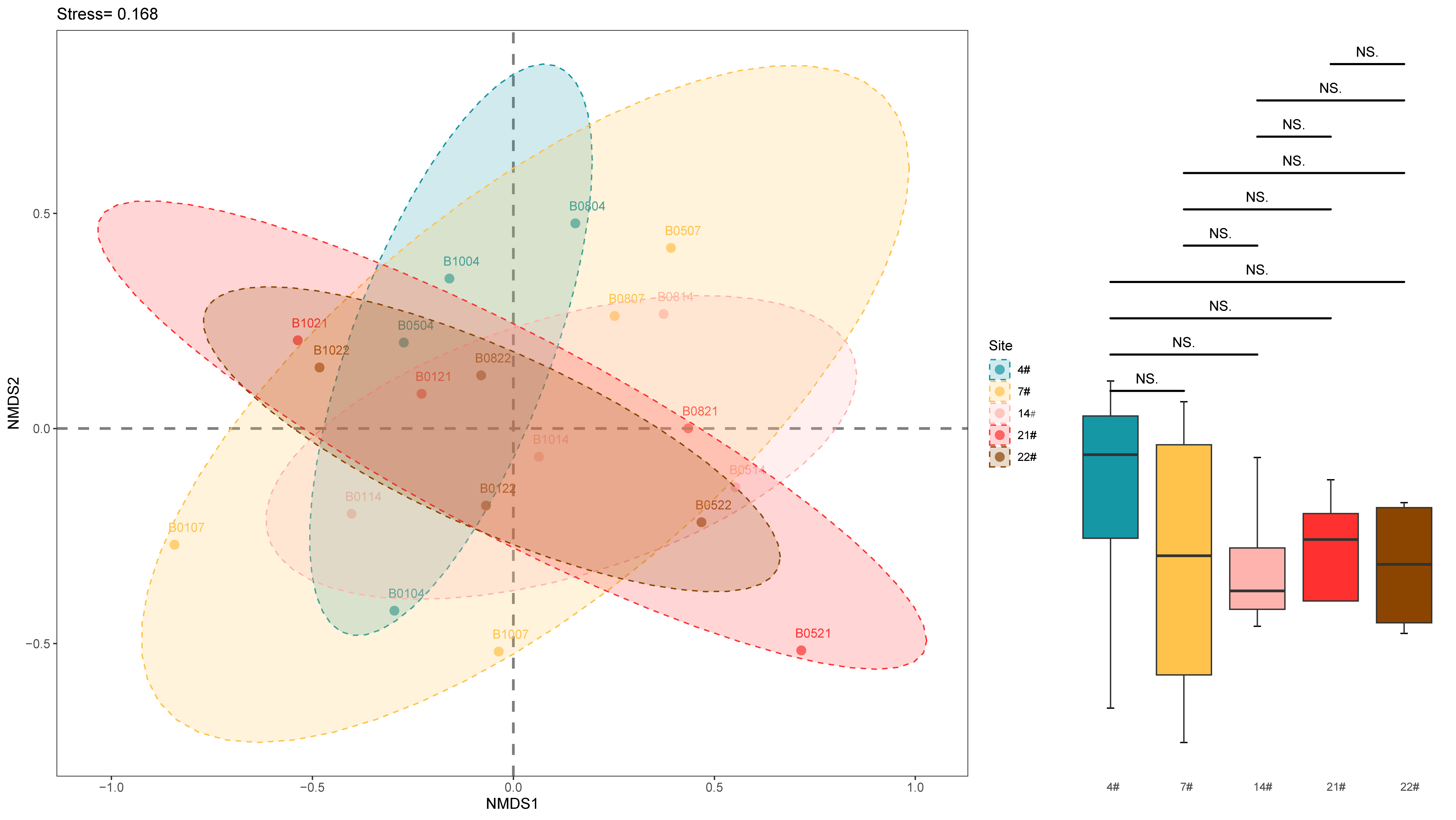

Supplement: Supplementary file 7 [file Image_3.jpeg]

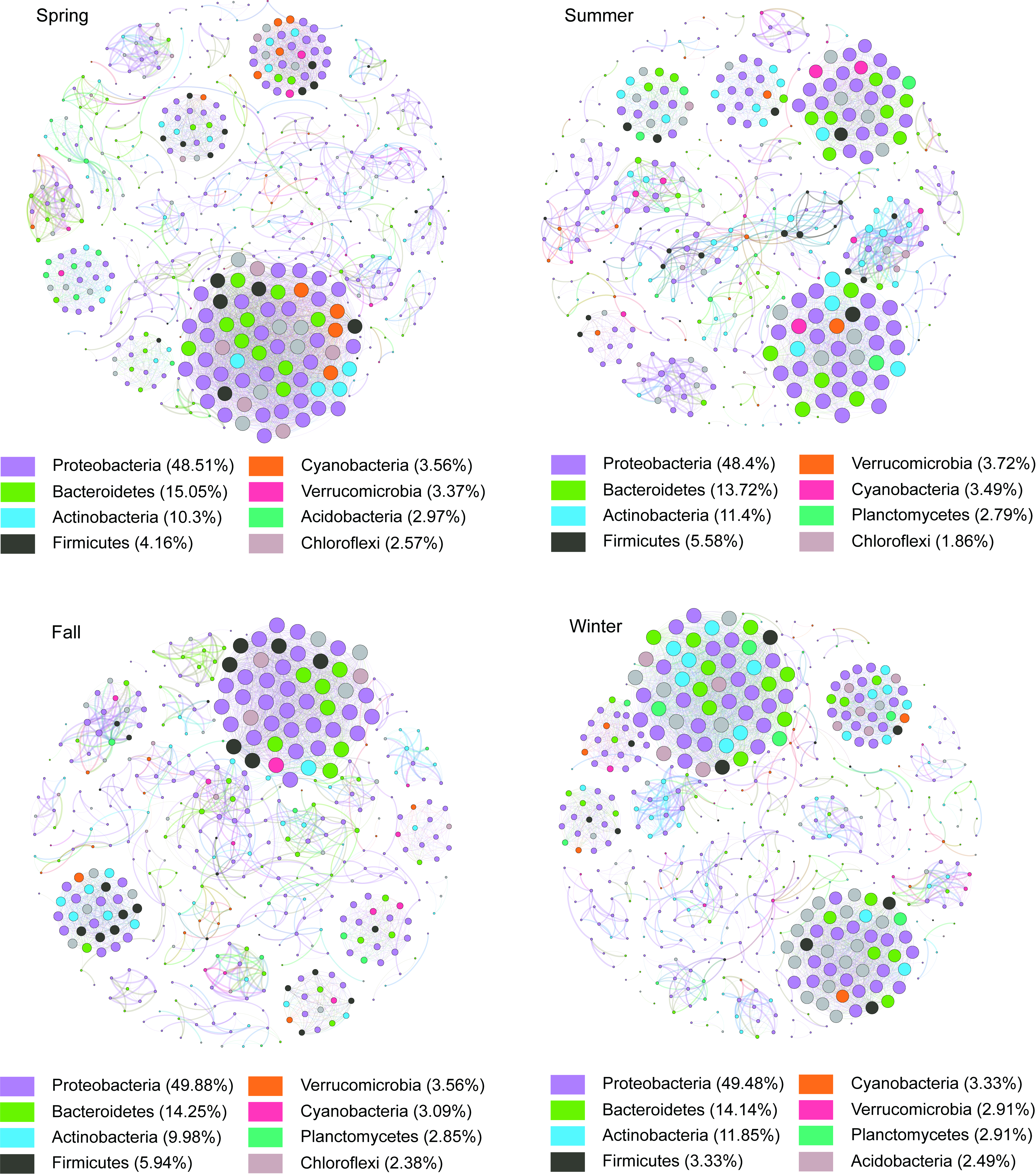

Supplement: Supplementary file 8 [file Image_4.jpeg]

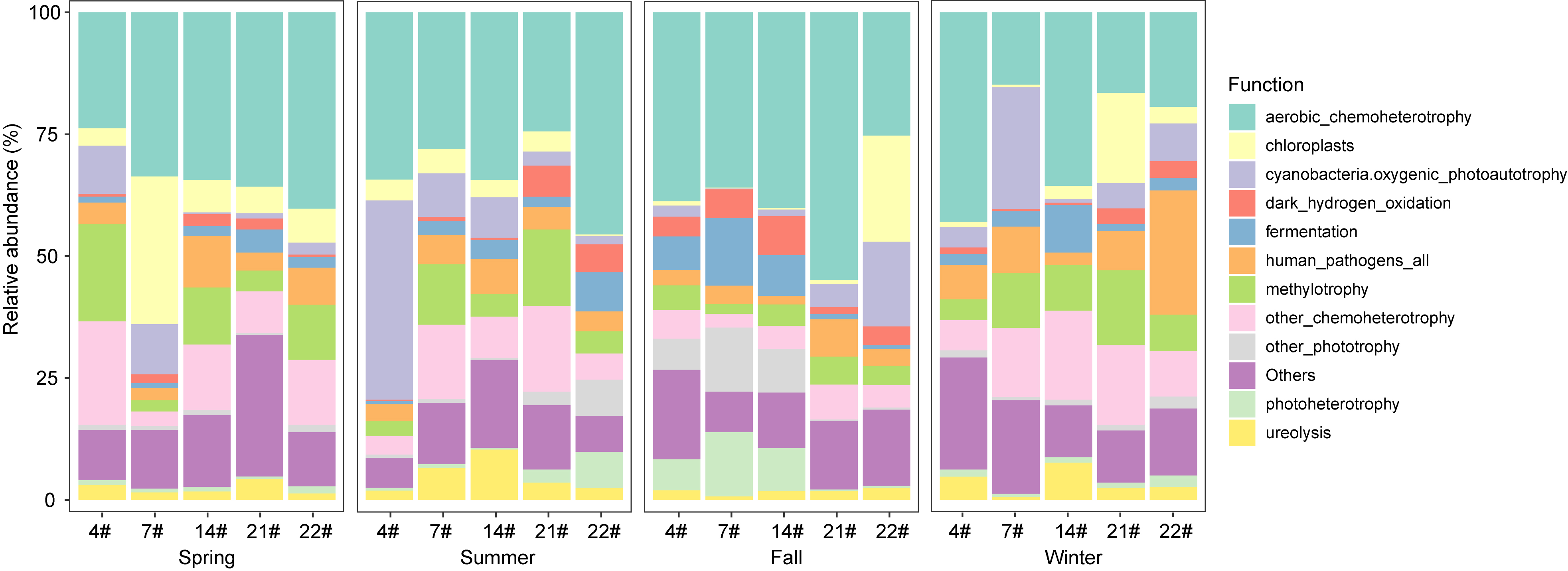

Supplement: Supplementary file 9 [file Image_5.jpeg]
